# TRACE: A Topological Algorithm for Detecting Additive-Coordinated Hydrate Cages

**DOI:** 10.1021/acs.jctc.5c01459

**Published:** 2025-11-14

**Authors:** Jun-Wei Hsu, Shiang-Tai Lin

**Affiliations:** Department of Chemical Engineering, 33561 National Taiwan University, Taipei 106319, Taiwan

## Abstract

Clathrate hydrates are crystalline inclusion compounds with relevance to global climate mitigation, energy resource development, and carbon capture and storage (CCS) technologies, owing to their high volumetric gas density and thermodynamic stability under moderate pressure–temperature conditions. The fundamental building blocks of hydrate crystals are polyhedral cages formed by hydrogen-bonded water molecules. Accurate identification of these cage structures in molecular simulations is critical for investigating the mechanisms of hydrate nucleation and growth. In this work, we introduce a novel open-source algorithm, TRACE (Topological Ring and Additive-Coordinated Cage Explorer), designed to detect and classify structural motifs, such as rings, cups, and both complete and incomplete cages, formed during hydrate nucleation in molecular dynamics simulations. A distinguishing feature of TRACE is its capability to include additives (e.g., hydrate formation promoters or inhibitors) in the cage identification process, making it particularly well-suited for investigating additive effects on hydrate formation. We validate TRACE by analyzing CO_2_ hydrate systems in the presence of urea, a known kinetic promoter. The algorithm enables the tracking of structural evolution and allows for the quantification of cage statistics, lifetimes, urea retention times, and nucleation kinetics via mean first-passage time (MFPT) analysis. By bridging structural and kinetic information, TRACE provides new insights into additive-modulated nucleation pathways and offers a robust tool for characterizing microstructural development in clathrate hydrate formation.

## Graphical abstract

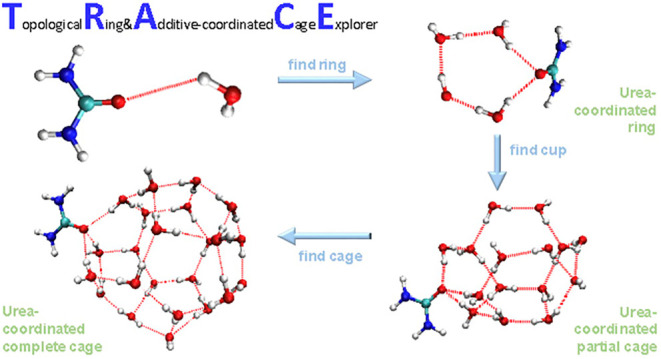

## 1 Introduction

Clathrate hydrates are crystalline compounds formed when water molecules organize into cage-like structures to encapsulate small guest molecules such as methane and carbon dioxide.^1,2^ The formation of clathrate hydrates has garnered significant research interest due to their relevance in natural phenomena (e.g., climate change and icy bodies in the solar system),^3–7^ their potential for technological applications (e.g., energy storage, seawater desalination, gas separation),^8–18^ and their impact on flow assurance in oil and gas transportation systems.^19,20^ In these contexts, the use of additives^21–31^ plays a critical role in modulating hydrate nucleation and growth dynamics. Understanding the molecular-level mechanisms by which these additives promote or inhibit hydrate formation is essential for the rational design of next-generation hydrate inhibitors and promoters. Many commonly studied additives, including methanol,^22^ l-methionine,^24^ leucine,^23^ and urea,^21,26,27,29^ are capable of forming hydrogen bonds with water molecules, thereby altering local hydrogen-bond networks. However, relatively few studies have systematically examined how such additives influence the local water structure during the early stages of nucleation.

Molecular dynamics (MD) simulations are a powerful approach for elucidating the mechanisms of clathrate hydrate nucleation at the molecular level. To quantitatively track the emergence of local structural ordering during hydrate formation, researchers commonly employ scalar order parameters such as F3, F4, and MCG.^32,33^ Complementary to these metrics, several algorithms have been developed to identify topological features of hydrate structures (i.e., rings, intermediate motifs, cages), including FSICA,^34^ GRADE,^35^ ICO,^36^ CHILL+,^37^ HTR and HTR+ frameworks.^38,39^ The strengths and limitations of these methods have been recently reviewed in reference.^40^

While these tools have advanced our understanding of hydrate structure, most are optimized for ideal, crystalline hydrate phases and tend to perform poorly in detecting disordered or amorphous intermediates due to limited structural tolerance or computational inefficiencies. This presents a significant challenge, as several leading nucleation models, including the blob formation and cage adsorption mechanisms,^41,42^ suggest that hydrate nucleation initiates with the assembly of transient, noncrystalline motifs. Moreover, many algorithms (e.g., FSICA, GRADE, ICO, HTR) focus primarily on identifying smaller ring structures (e.g., 4-, 5-, and 6-membered rings), even though early nucleation events often involve larger, less ordered rings, such as 7-, 8-, or even 10-membered configurations. Furthermore, most existing algorithms do not account for hydrogen bonding between water and molecular additives. This limits their applicability for studying additive-mediated nucleation, where additives (e.g., urea or amino acids) may interact with the water hydrogen-bond network to stabilize or destabilize the formation of noncanonical cage structures. Addressing these gaps is essential for gaining a complete picture of the molecular pathways involved in both homogeneous and heterogeneous hydrate nucleation.

In this work, we present a new open-source algorithm, TRACE (Topological Ring and Additive-Coordinated Cage Explorer), which integrates the key advantages of existing topology-based methods while allowing users to incorporate user-defined water–additive hydrogen bonding. TRACE is designed to track complex topological motifs, including larger-membered rings, cups, and both incomplete and complete cages formed through additive–water coordination, thereby facilitating detailed analysis of additive–water interactions during hydrate formation. By following specific molecular trajectories, TRACE enables the quantification of cage populations, cage lifetimes, and the residence time of additives such as urea near hydrate cages. Nucleation dynamics and cluster evolution can thus be analyzed using a mean first-passage time (MFPT) framework, enabling the extraction of key nucleation parameters in the classical nucleation theory (CNT).

## 2 Method

TRACE is an open-source C++-based program and is freely available on GitHub.^43^ TRACE integrates and extends core features from several established hydrate topology analysis methods, including GRADE,^35^ ICO,^36^ and HTR,^38^ while introducing several conceptual and algorithmic advancements. From GRADE, TRACE adopts geometric ring descriptors for efficient identification of polygonal ring structures. TRACE employs an iterative topology-based approach that builds upon the concept of partial cage-like motifs, or “cups,” originally introduced in ICO, but with key modifications tailored for amorphous systems. Moreover, TRACE implements a classification framework—also derived from ICO—that distinguishes between standard edge-saturated cages (SECs) and nonstandard edge-saturated cages (non-SECs), enabling a more comprehensive characterization of diverse cage morphologies. A comparison of cage definition used by different algorithms is provided in Section 4 of the Supporting Information.

To enhance computational efficiency, TRACE adopts and adapts the spatial partitioning strategy originally developed in HTR for large-scale particle systems, optimizing it for the molecular topologies encountered in hydrate nucleation.

Incomplete cages are identified based on a customized set of topological criteria tailored to recognize structures that are geometrically close to complete cages. This approach contrasts with the FSICA method, which employs edge-saturated indices (ζ_V_) and face-saturated indices (ζ_E_).^34,44^ In FSICA, a polyhedron-like cage structure that satisfies both the edge-saturation and face-saturation conditions is identified as a complete cage (CC); otherwise, it is designated as an incomplete cage (IC). Since the number of possible IC configurations increases combinatorially, exhaustive enumeration may become computationally prohibitive. To address this challenge, TRACE focuses on the efficient and selective detection of structurally plausible intermediates that are mechanistically relevant to hydrate formation. The detailed algorithm for IC detection is described in Section 2.4.

Figure 1 presents an overview of the TRACE workflow and delineates the computational procedures implemented to identify cage structures in hydrate-forming systems. The process begins with parameter initialization and input file verification, followed by an optional step to define hydrogen bonding criteria involving additives. For each simulation frame, atoms are spatially partitioned into grids to efficiently construct hydrogen-bond maps and neighbor lists.

**Figure 1:**
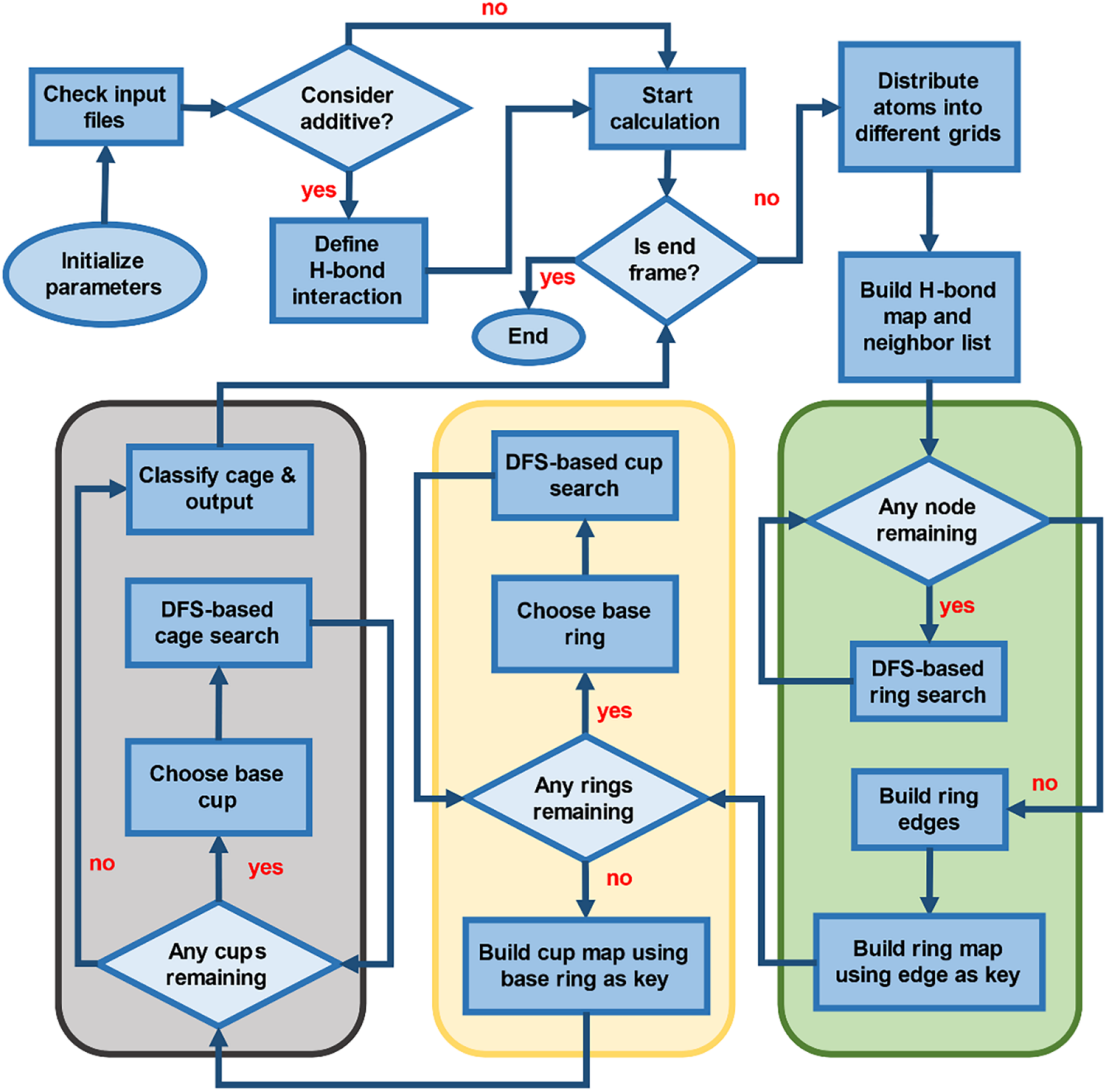
Structure and workflow of TRACE for identifying hydrate-relevant cage structures in MD simulations.

Ring structures are identified using a depth-first search (DFS)-based algorithm. For each ring, directed edges are consistently constructed in either a clockwise or counterclockwise orientation, ensuring that duplicate rings traversed in the opposite direction are filtered out. Each edge is represented by the ordered vertex pair (min­(a, b), max­(a, b)) to ensure uniqueness and prevent redundant edge definitions. These edges are stored as keys in a hash map, allowing efficient retrieval of all rings sharing a given edge for subsequent cup construction.

The identified rings form the basis for constructing base and lateral rings, which are subsequently combined into cups (as shown in the yellow box in Figure 1), intermediate motifs that serve as building blocks for more complex cages. Each cup’s base ring is stored as a hash map key to facilitate rapid searching of compatible cups that share the same base ring, enabling efficient cage construction. Finally, TRACE classifies cage structures as complete or incomplete (gray box) based on topological criteria. Throughout the workflow, TRACE maintains atom-level identity tracking and supports hydrogen bonding between water and additives, allowing for detailed structural and kinetic analyses across simulation trajectories.

### 2.1 Hydrogen Bond Identification

The geometric criteria of Luzar and Chandler^45^ are adopted for identifying hydrogen bonds between water molecules. The geometric criteria consist of two conditions, as illustrated in Figure 2: (1) The distance between donor and acceptor oxygen atoms, *r*_da_, should be less than a threshold *r*_cut_. (2) The angle formed by the donor-hydrogen, donor-oxygen and acceptor-oxygen, θ_da_, must be less than θ_cut_. A typical hydrogen bond is identified if *r* < 0.35 nm and θ < 30° under ambient conditions, while adjustments to the threshold values for r and θ are commonly seen in different studies.^38,46^

**Figure 2:**
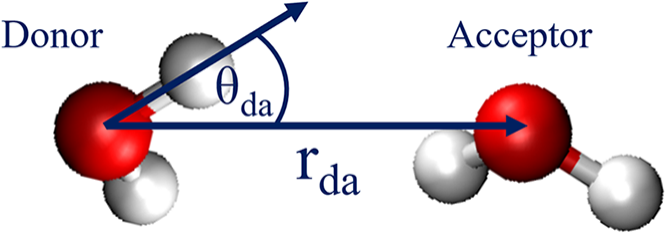
Schematic representation of geometric criteria for hydrogen bond: *r*_da_ ≤ *r*_cut_ and θ_da_ ≤ θ_cut_.

To efficiently construct hydrogen bond information in a sparse graph, we use a hash map keyed by molecule pairs, where values encode bonding status: zero indicates no bond, and positive integers represent bonded atom identities, preserving atom-level detail. Each molecule maintains a neighbor list of bonded molecules sorted by their molecular indices in ascending order, which is updated every simulation frame, resulting in a substantial computational cost. To reduce this, the simulation box is partitioned into a 3D spatial grid, assigning molecules to cells based on coordinates. Molecules within a cutoff distance *r*_cut_ of a cell boundary are also assigned to adjacent cells, and those near box boundaries are assigned across periodic boundaries to maintain interaction continuity. This limits hydrogen bond searches to molecules within the same cell, avoiding a full 3 × 3 × 3 neighborhood scan.

Figure 3 exemplifies this scheme in a 2D box divided into a 2 × 2 grid. Atoms near grid boundaries (blue lines) are assigned to multiple adjacent cells to avoid missing hydrogen bond pairs that cross cell boundaries. For example, atom 9, originally in grid [1][1], is also assigned to grids [0][1], [0][0], and [1][0]. Atoms near the box origin within *r*_cut_ (e.g., atom 0 in grid [0][0]) are similarly duplicated across PBC to grids [0][1], [1][0], and [1][1]. The gray inset illustrates final atom assignments: black labels indicate atoms originally in each grid, while red labels mark duplicated atoms due to proximity to boundaries. The same assignment procedure applies to additives with multiple hydrogen bond donor or acceptor sites. For multiatomic molecules with multiple atoms in the same grid cell, the algorithm prevents redundancy by not reassigning a grid cell once it has been assigned to that molecule.

**Figure 3:**
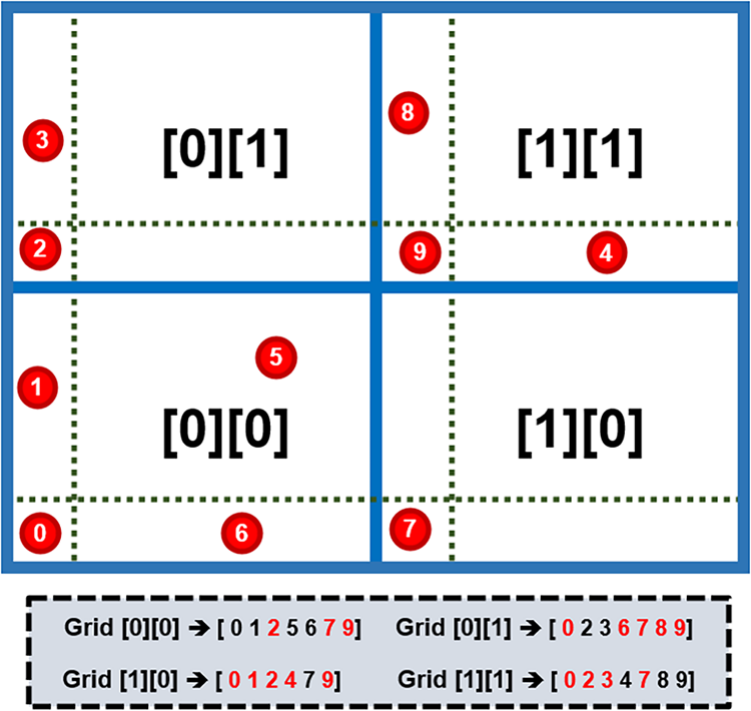
Schematic of atom distribution in 2-D grid cells. Red atoms represent H_2_O oxygens with molecule residue ID. Gray boxes show H_2_O considered for each grid; black numbers indicate molecules originally inside the grid, and red numbers indicate molecules duplicated due to PBC or buffer regions.

### 2.2 Ring Structure Identification

Ring structures are identified using a depth-first search (DFS) algorithm for its simplicity, inherent path-order preservation, and easy pruning. TRACE treats each molecule—H_2_O or additive—as a single node. Small rings (4–6 members) require no further processing, while larger rings (7–12 members), often formed by complex composites (e.g., 5 + 6, 5 + 5 + 5), are filtered using two geometric criteria: each vertex must form exactly two hydrogen bonds with neighbors along the DFS path, and the ring must satisfy an interior angle tolerance. This efficiently captures large rings while excluding most composite rings, because hydrate cages are primarily composed of near-regular polygons.

Given that the search space increases rapidly with ring size, geometric and physical constraints are employed to prune the DFS tree and accelerate detection without compromising accuracy. Specifically, at DFS level 2 (when three atoms are connected), a reference plane is defined using their positions. For each subsequent level i, the dihedral angle, ϕ, between the reference plane and the plane formed by atoms (i, i–1, i–2) is evaluated. If the angle exceeds a user-defined tolerance ϕ_t_, the path is discarded.

1 $$\phi <\phi_{t}\;\left(\mathrm{accepted}\right)$$

For 7-membered and larger rings, additional pruning is applied based on interior angles, θ_IA,i_. Each interior angle along the path is compared to that of a regular n-gon, and any deviation exceeding an angle tolerance (θ_IA,t_, typically between 20–30°) results in path rejection.

2 $$\left|\theta_{\mathrm{IA},i}-\frac{(n-2)\times 180^{{}^{\circ}}}{n}\right|\leq \theta_{\mathrm{IA},t}\;\left(\mathrm{accepted}\right)$$

The choice of this angular tolerance (θ_IA,t_) significantly affects detection accuracy. A large interior angle tolerance results in more misidentification of large fused rings (e.g., 5 + 6, 6 + 6), whereas a tolerance that is too small (below 20°) tends to underdetect 7 and larger rings. Therefore, we recommend the use of 20° as the interior angle tolerance. In the interior angle and dihedral angle analysis, urea is considered as a single node, whose location is determined as the geometric mean of the urea atoms involved in the topological analysis. (See Figure S5 in the Supporting Information for further details)

From 7-membered rings onward, ring closure is further verified to avoid pseudorings across periodic boundaries, which are rare but a possible issue in small systems containing long-chain additives.

Figure 4 illustrates the identification process of a valid six-membered ring, [0, 1, 2, 5, 10, 11], using a depth-first search (DFS)-based ring detection algorithm. The search begins at atom 0 and proceeds through atoms 1 and 2, where a reference plane is defined based on their coordinates. When attempting to extend the path to atom 3, the algorithm evaluates the dihedral angle ϕ between the reference plane (atoms 0–1–2) and the new plane formed by atoms 1–2–3. As this dihedral angle exceeds the user-defined dihedral angle tolerance (ϕ_t_), the algorithm prunes all subsequent paths that would proceed through atom 3. The search then backtracks to atom 2 and continues along an alternative branch to atom 5, where the dihedral angle between planes (1–2–5) falls within the tolerance; subsequent dihedral angles along this path are assumed valid for clarity.

**Figure 4:**
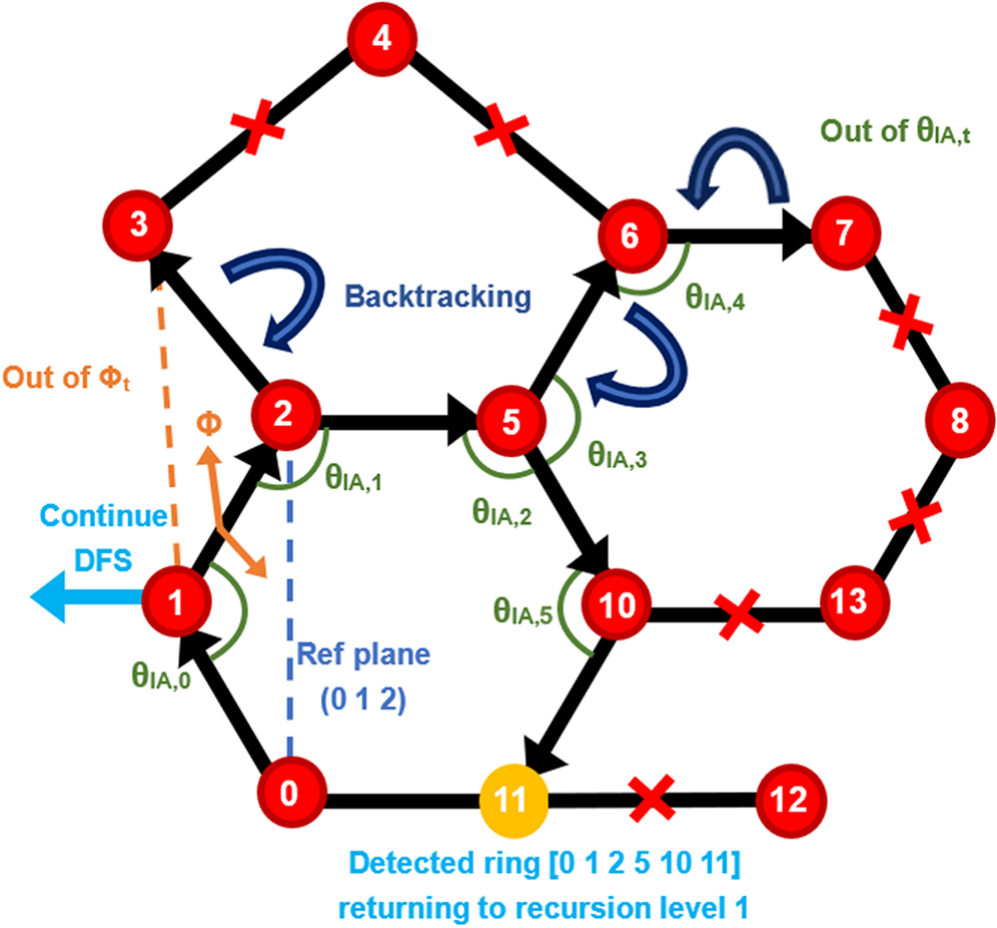
Illustration of the depth-first search for ring detection. Nodes represent molecules (additive or water), with the search proceeding from smaller to larger residue IDs. Dihedral and interior angles are calculated every three consecutive nodes; violations trigger backtracking. For paths ≥ 6, all interior angles are checked before advancing. The search completes a ring at node 11 and then backtracks to node 1 to continue the DFS while avoiding redundancy.

The path then extends to atom 7, and before proceeding to atom 8, the algorithm evaluates all interior angles along the current path (θ_IA,0_ through θ_IA,5_). As not all of these angles fall within the predefined tolerance—determined based on the assumption that a 7-membered ring may form at the next level (i.e., the current path length is 6)—the path is considered geometrically distorted and is discarded, along with all downstream nodes connected to atom 7. The algorithm then backtracks to node 5, and the corresponding interior angle entries (e.g., θ_IA,3_ and θ_IA,4_) are removed from the interior angle cache. The algorithm then explores the branch from atom 5 to 10, and subsequently to atom 11 (visited before atom 13 due to index order). Upon reaching atom 11, which is directly bonded to the starting atom 0, a closed loop is formed. As the ring is already complete, the algorithm neither extends the path to other neighbors (e.g., atom 12) nor backtracks to atom 10 to explore alternative ring closures, and instead returns to level 1 (atom 1) to search for other potential paths. This candidate ring satisfies all structural and topological criteria—planarity, bounded angular distortion (noting that only n–2 interior angles and n–3 planes are evaluated for an n-membered ring, rather than all n interior angles or C­(n,3) dihedral angles) accepted as a valid six-membered ring. After exploring all paths originating from atom 0, the node is permanently locked to prevent redundant searches and enhance computational efficiency. While this strategy may exclude some rings that share more than two vertices with already-detected rings, these highly distorted or topologically complex structures typically do not represent physically relevant hydrate cages. In practice, such configurations do not contribute to meaningful hydrate structures but would unnecessarily complicate subsequent combinatorial searches for cages and reduce algorithmic efficiency. Figure 5 illustrates examples of different size rings and urea-coordinated rings.

**Figure 5:**
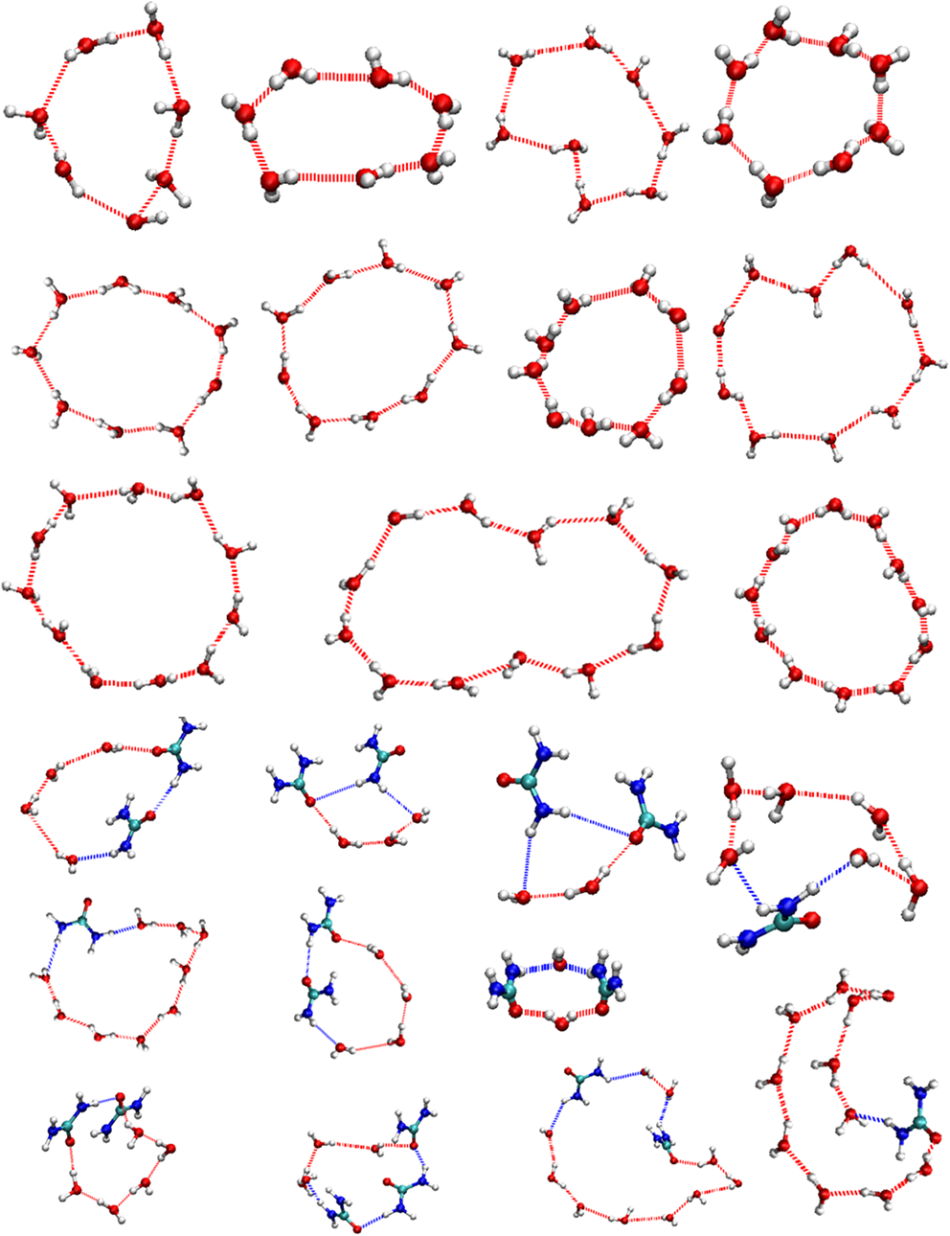
Representative ring structures identified by TRACE. Top three rows illustrate water-only rings and the bottom three rows are urea-coordinated rings.

### 2.3 Cup Structure Identification

Cup structures and lateral rings are identified following the GRADE framework.^35^ A cup consists of rings connected to a central base ring by sharing exactly one edge. Detection begins with a selected base ring, and all rings sharing one edge with it are considered lateral neighbors. A depth-first search (DFS) is employed to explore possible lateral sequences: at each step, candidate rings not sharing an edge with the immediately preceding ring are pruned. A cup is valid only if the final lateral ring shares edges with both the preceding and first lateral ring, ensuring closure. Each base ring can theoretically form up to two cups oriented in opposite directions, though more may occur in highly distorted structures.

Figure 6 illustrates the DFS procedure for identifying a cup structure originating from a four-membered base ring (shown in the top-left). The algorithm begins by selecting the first lateral ring, which must share the first edge of the base ring. This ring is designated as the first lateral ring at level 0 in the DFS tree (top row, middle). The search then attempts to find a second lateral ring at level 1. In the first attempt (top row, right), the candidate lateral ring does not share an edge with the first lateral ring, violating the adjacency condition required for a cup structure. Therefore, the algorithm backtracks to level 0 and selects an alternative candidate. The new choice for the second lateral ring correctly shares an edge with the first lateral ring, allowing the search to continue (middle row, left and middle). At level 2, the algorithm proceeds to add a third lateral ring. Initially, an invalid candidate is encountered (middle row, right), which again prompts a backtrack to level 1. A valid third lateral ring is then selected (bottom row, left), maintaining edge connectivity with the second lateral ring. Finally, at level 3, the algorithm identifies a fourth lateral ring (bottom row, right) that shares edges with both the third lateral ring and the first lateral ring, thereby completing a closed cup topology. This recursive search procedure ensures that each lateral ring is edge-connected to its predecessor and that the final lateral ring closes the cup by bridging back to the starting ring.

**Figure 6:**
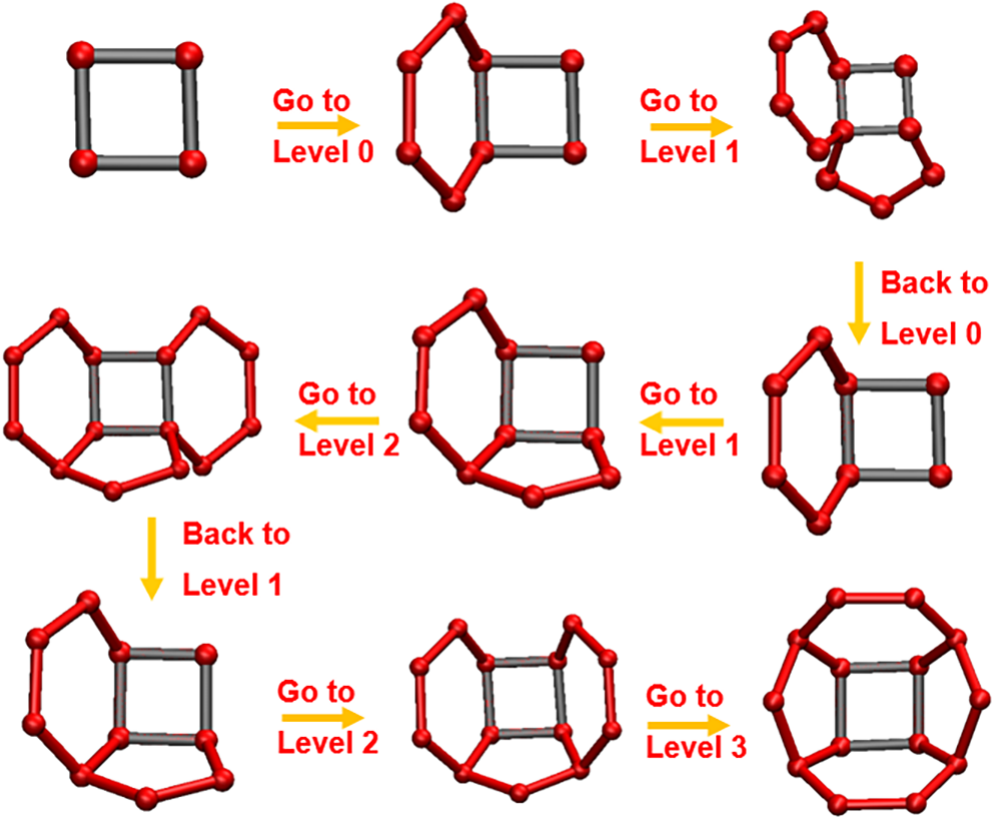
Depth-first search for cups (top view). Starting from a base ring, lateral rings are sought following edge order. Each new lateral ring must be adjacent to the previous one; if not, backtracking occurs. The process continues until all lateral rings are found, with the last ring completing the cup by adjoining both the first and previous rings.

### 2.4 Cage Structure Identification

To identify cage structures, we extend the iterative cup-overlapping approach from ICO [51] with a refined DFS strategy. Figure 7 illustrates an example of an incomplete cage topology originating from a base cup. The base ring defines layer 1, while the initial set of lateral rings, which share edges with the base, constitute layer 2 (highlighted in red). Each lateral ring in layer 2 serves as a parent ring, providing a reference for locating additional adjacent cups. The algorithm proceeds by overlaying cups connected to these parent rings. By systematically removing shared faces from overlapping cups, the algorithm identifies new rings (shown in cyan), which represent the next layer of lateral rings, referred to as layer 3. This recursive topological expansion continues until all lateral rings at the current layer have been explored. In the specific case shown, five new lateral rings are generated at layer 3, each of which is recorded as a parent ring for potential further growth in subsequent iterations. This hierarchical approach enables systematic identification of both complete and incomplete cage structures, capturing complex topologies relevant to hydrate nucleation.

**Figure 7:**
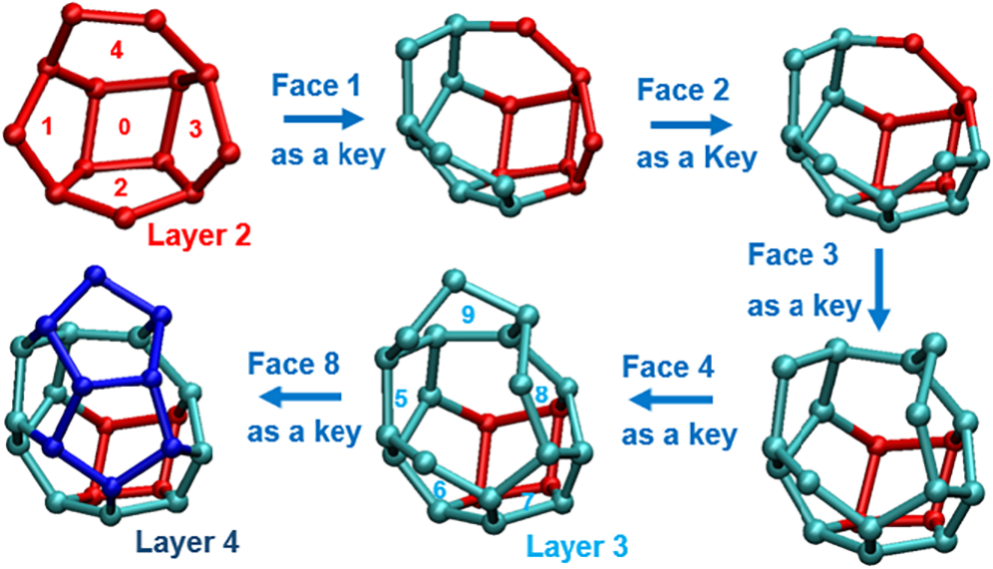
Schematic representation of the depth-first search (DFS) algorithm used to detect a 4^1^5^8^6^4^ incomplete cage (top view). The search starts from a base cup (red). Each lateral ring (labeled 1–9) recursively explores an associated cup, where the nonoverlapping portion (excluding the intersection with the existing structure) defines a new lateral ring. The process continues iteratively until a termination condition is satisfied.

To avoid combinatorial explosion, we adopt a heuristic approach that collectively processes all available lateral rings at each layer rather than exhaustively enumerating all possible ring subsets. While this strategy may overlook some optimal or alternative configurations, the impact is minimal because the omissions predominantly involve incomplete cages (ICs) and do not compromise the identification of complete cages. This trade-off provides an effective balance between accuracy and computational efficiency.

Applying the same approach, layer 4 is constructed using the five cyan lateral rings identified at layer 3. During this process, multiple candidate cups with different orientations may emerge, which could lead to combinatorial explosion if unchecked. To prevent this, we enforce a parent-ring constraint, requiring that each new candidate cup must include its designated parent ring. This condition is applied consistently throughout the topology expansion: starting at layer 2, where the base ring acts as the parent for all initial lateral cups, and continuing through higher layers, where newly generated rings recursively assume the role of parent for their subsequent lateral expansions. As illustrated in Figure 7, this approach allows the topology to grow to layer 4 (blue rings), meets the defined termination criteria, and identifies the structure as a valid incomplete cage.

It is also worth noting that each additive molecule is treated as a node during cage construction. Therefore, each vertex in the schematic shown in Figure 7 may represent either an additive or a water molecule. Furthermore, if a molecule forms multiple hydrogen bonds with another molecule, TRACE represents these multiple interactions as a single edge in the cage graph, thereby avoiding redundant connectivity in the network representation.

TRACE first identifies SECs, after which their associated cups are removed from the search pool to prevent duplication. The algorithm then proceeds to detect ICs. Due to the combinatorial complexity of partial cage motifs, many similar IC candidates may arise. To manage this, once cups associated with an IC are assigned, they are immediately excluded from further consideration (a “first-come, first-served” strategy). This approach prioritizes earlier-detected ICs and minimizes redundancy, balancing completeness with computational efficiency.

To enhance search efficiency, we introduce the concept of theoretical isolated vertices (T) to facilitate the termination of cage search. For a graph consisting of arbitrary polygons without shared vertices, edges, or faces, T is defined as the sum of all vertices of each face:

3 $$T=\sum_{n}V_{n}$$

where n is the total number of faces of a cage, and V_n_ is the number of vertices of the n^th^ face. For a SEC, two conditions must be met: each vertex is shared by exactly three edges, and each edge is shared by exactly two faces. Therefore, the expected total numbers of vertices and edges are T/3 and T/2, respectively. This definition of T avoids tracking the full topology during DFS. It also enables tolerance-based identification of ICs, which traditional Euler characteristic methods (eq 4) cannot easily accommodate.

4 face−edge+vertices=2

During the recursive depth-first search (DFS), each search path is either accepted as a valid cage or pruned based on the following criteria:

(1) For complete cages (SECs or non-SECs), if the number of distinct vertices found is less than or equal to T/3; for incomplete cages (ICs), this threshold is relaxed to (T + 2)/3.
(2) The search depth reaches layer 4.
(3) No lateral rings remain to explore.
(4) The number of path combinations at the current layer exceeds 10.

If a path satisfies condition (1), it is immediately terminated as a valid candidate—classified as an SEC, non-SEC, or IC—and no further exploration is conducted. If condition (2), (3), or (4) is met, the current path is pruned and the search backtracks to explore alternative DFS branches. In other cases, the search continues to the next depth layer. This recursive pruning strategy ensures the identification of topologically valid motifs while maintaining computational efficiency.

Finally, the full topology of each cage candidate is constructed, including its vertices, edges, faces, and their connectivity. SECs and non-SECs are identified according to the ICO definition. For ICs, we restrict the search to structures that are close to complete cages. To enforce this, additional filtering criteria are applied to retain only those ICs that closely resemble SECs. Specifically, candidate ICs must satisfy the Euler characteristic and ensure that each edge is shared by exactly two faces and each vertex is shared by at least two edges. These ICs are a subset of the face-saturated incomplete cages (FSICs). Note that, in FSICA, a ring with 7 and more members is considered as a hole, whereas in TRACE, larger rings are allowed when searching both for complete cages and for incomplete cages. Figures 8, 9, and 10 show representative examples of complete cages (CCs), incomplete cages (ICs) and urea-coordinated hydrate cages identified by our algorithm.

**Figure 8:**
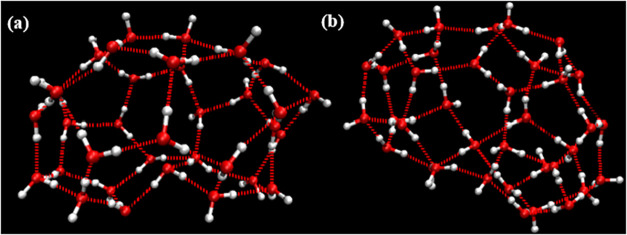
Distorted nonstandard edge-saturated cage and incomplete cage structures: (a) 4^1^5^13^6^5^7^1^ and (b) 4^1^5^14^6^1^7^4^.

**Figure 9:**
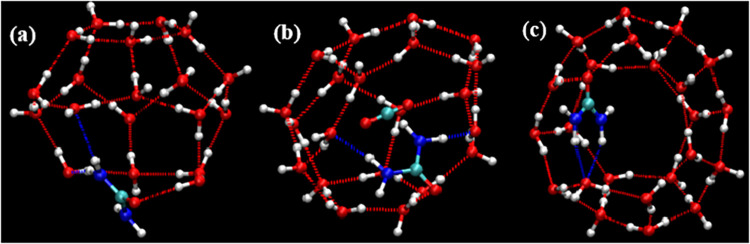
Schematic representations of urea-coordinated SECs: (a) 5^12^ (b) 4^1^5^10^6^2^ cage (with a guest molecule) (c) 5^12^6^2^ cage.

**Figure 10:**
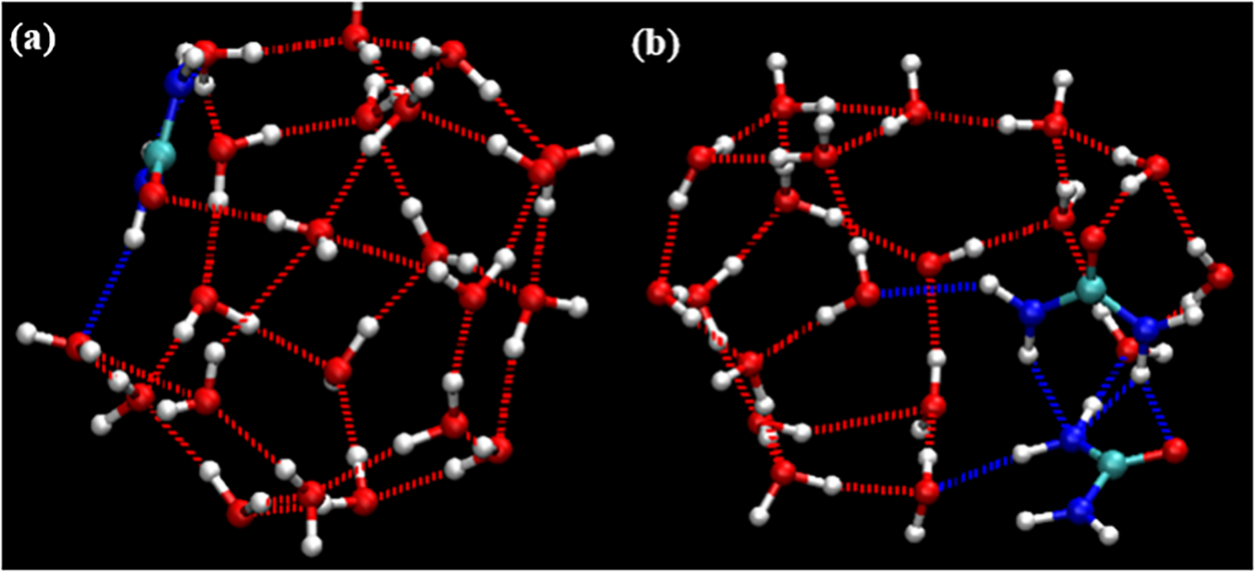
Schematic representations of urea-coordinated cages: (a) A 4^2^5^10^6^2^ non-SEC structure. (b) A 4^2^5^6^6^4^ incomplete cage structure.

Once all SECs, non-SECs, and ICs have been identified, cage clusters are determined based on face sharing. Specifically, cages that share at least one face are grouped into the same cluster, allowing us to capture their spatial connectivity and potential cooperative behavior.

### 2.5 Identification of Caged Guest Molecules

A centroid-based method is employed to identify caged guest molecules. A guest is classified as encapsulated if its distance to the cage’s geometric centroid falls below a face-number-dependent threshold: < 0.05 nm for cages with ≤8 faces, <0.1 nm for 9–10 faces, <0.2 nm for 11–16 faces, and <0.35 nm for >16 faces. To increase efficiency, cages with ≤16 faces are restricted to a single guest molecule, while larger cages permit multiple occupancy. Although this distance-based criterion is less rigorous for highly distorted cages, it performs well in the vast majority of cases, offering a good balance of computational efficiency and robustness, especially when applied to disordered or amorphous structures. Representative examples are shown in Figures 11, 12, 13, and 14.

**Figure 11:**
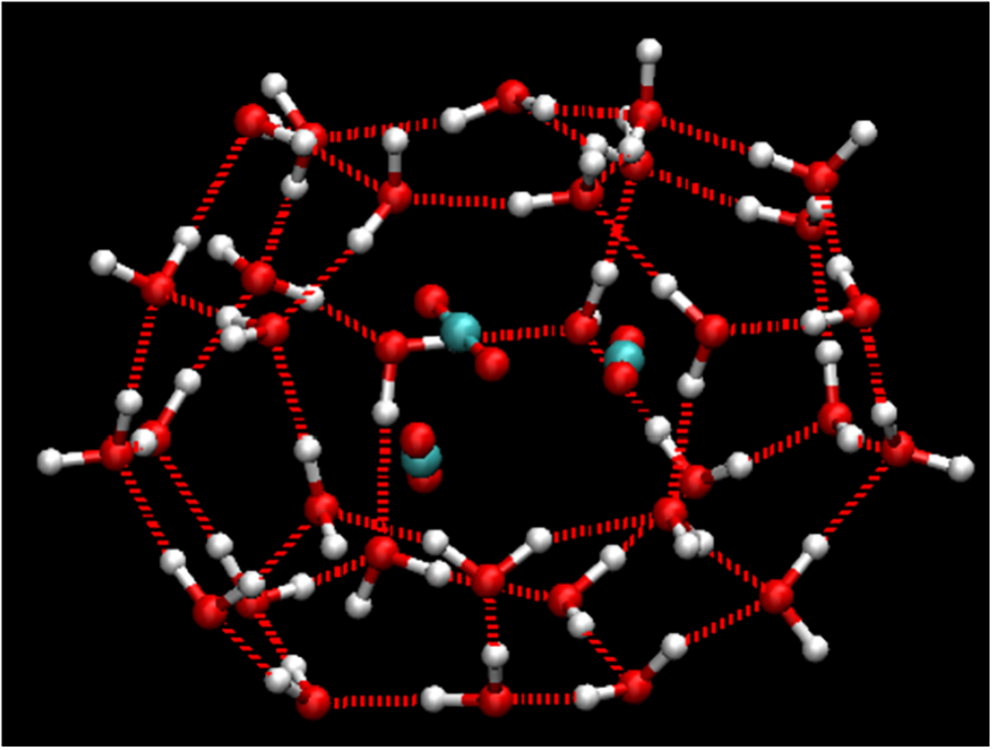
Schematic diagram of a 4^1^5^11^6^5^7^1^ standard edge-saturated cage containing three guest molecules.

**Figure 12:**
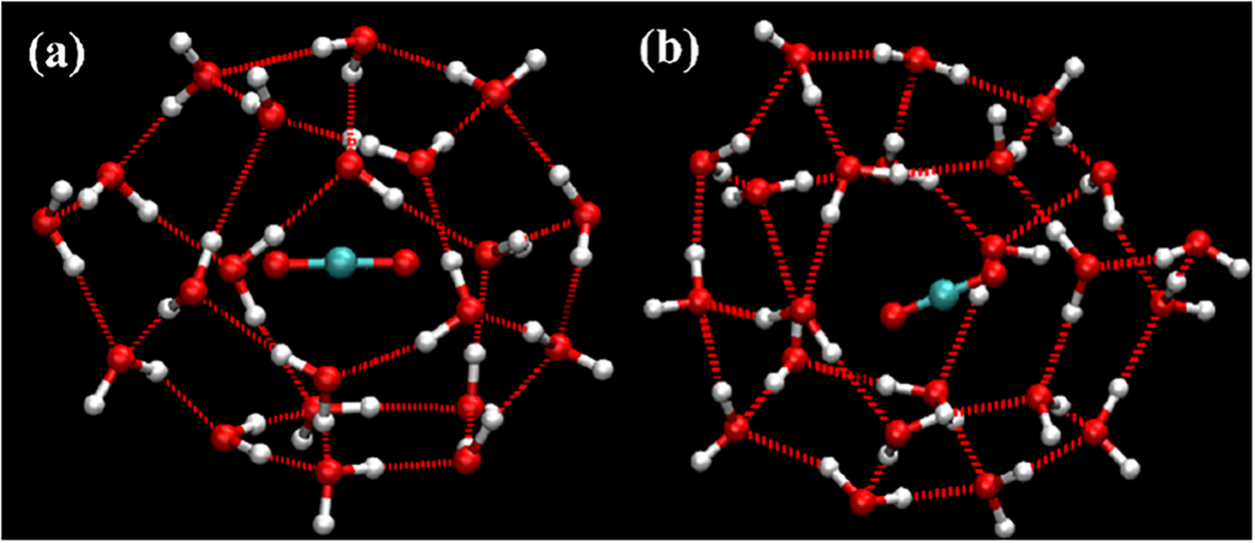
Schematic diagram of incomplete cages: (a) 5^10^6^2^, (b) 5^10^6^3^.

**Figure 13:**
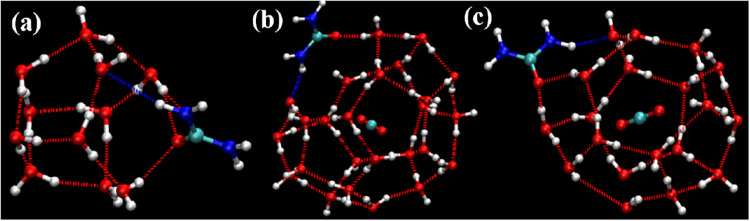
Schematic diagrams of urea-containing cages: (a) 4^4^5^4^ (SEC) without guest, (b) 4^2^5^8^6^2^ (SEC) with guest, and (c) 5^12^6^3^(SEC) with guest.

**Figure 14:**
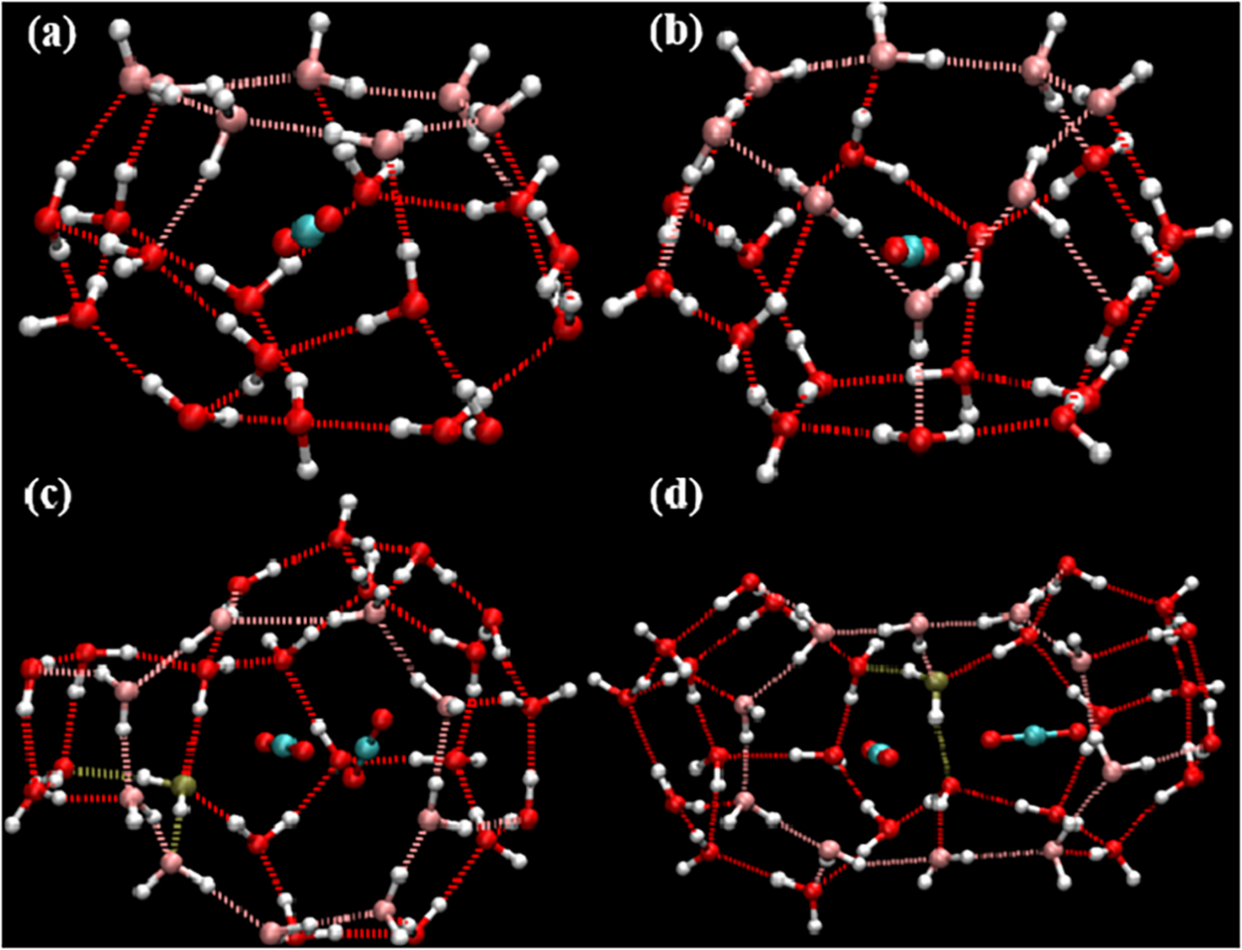
Schematic diagram of complete cages: (a) 4^3^5^7^6^2^7^1^ (SEC), (b) 4^3^5^8^6^2^8^1^ (SEC), (c) 4^5^5^9^6^3^9^1^ (non-SEC) and (d) 4^2^5^16^6^1^10^1^ (non-SEC). Oxygen atoms colored in pink (participating in 7–10-membered rings) and dark tan (shared by four faces) highlight their respective structural roles.

### 2.6 Crystallinity

The cage structures identified can be used to determine the degree of gas hydrate crystallinity of a nucleating system. Here we define the crystallinity index (CI) as the average number of hydrate cages in which each water molecule participates. It is given by

5 $$\mathrm{CI}=\frac{1}{4M_{\mathrm{tot}}}\sum_{M=1}^{M_{\mathrm{tot}}}n_{M}$$

where *M*_tol_ is the total number of water molecules, and *n*_*M*_ is the number of cages in which molecule *M* participates. In a perfect hydrate structure, each water molecule is involved in four hydrogen bonds (two via lone pairs and two via hydrogen atoms), which are edges of cages. Since different cages around a node can be differentiated using 3 edges, the water molecule is involved in four cages (*C*_3_^4^ = 4). By applying the normalization factor of 1/4, a fully crystalline hydrate exhibits a crystallinity index value close to 1. In contrast, disordered or liquid states have crystallinity values approaching zero. During nucleation, intermediate CI values indicate the degree of partial structural ordering.

## 3 Result and Discussion

### 3.1 Performance of the TRACE Algorithm

We performed a comprehensive benchmarking of TRACE to evaluate both accuracy and computational efficiency in identifying hydrate cages over a range of nucleation environments:

(A) L-H (Thick gas phase): A heterogeneous system with a thick CO_2_ vapor layer maintained throughout the nucleation process.
(B) H-L-V (Three-phase system): A system exhibiting coexistence of hydrate, liquid water, and CO_2_ vapor phases.
(C) L_CO_2__ (Supersaturated solution): A single-phase supersaturated (X_CO_2__= 0.1739) CO_2_–H_2_O solution.
(D) L-H (Thin gas phase): A heterogeneous system where the initially present thin CO_2_ gas layer fully dissolves into the liquid phase during nucleation.
(E) Perfect hydrate crystal: An ideal reference structure for verifying cage detection accuracy under crystalline conditions (The perfect clathrate hydrate structures were constructed based on Takeuchi et al.^54^ In this work, the sH hydrate unit cell was converted from two hexagonal unit cell.).

The phase labels used are L for the H_2_O liquid phase, L_CO_2__ for the CO_2_ solution phase, H for the hydrate phase, and V for the CO_2_ vapor phase. Additives (urea) are introduced to some of the systems (A1, A2, and B2. See Table 1) to examine the performance in finding urea-coordinated cages. Details of these structure generation are provided in the Supporting Information. All benchmarks were conducted on a dual-socket AMD EPYC 9124 system with 32 physical cores (16 cores per socket @ 3.0 GHz), running 64-bit Linux. Background processes were minimized to ensure consistent timing measurements. TRACE utilized close to 95–100% of a single CPU core, and all timing variances remained within 5%.

**Table 1:** Performance and Cage Detection Summary for Various Systems^a^

system	# H_2_O/CO_2_/urea	time (TRACE) [s]	time (HTR) [s]	# of SECs/non-SECs/ICs (TRACE)	# of urea-coordinated SECs/non-SECs/ICs (TRACE)	# of SECs (HTR)
*A1: L-V	1000/500/2	0.139	1.102	78/0/20	1/0/0	77
*B1: H–L–V	1840/320/0	0.115	1.079	81/0/5	0/0/0	80
*C1: L_CO2_	2944/512/0	0.419	2.258	216/4/54	0/0/0	188
*A2: L-V	3000/1500/50	0.291	1.899	11/0/15	0/1/0	10
*C2: L_CO2_	8832/1536/0	0.906	3.442	8/0/39	0/0/0	7
*D1: L-V	29440/5120/0	2.211	56.99	10/0/19	0/0/0	6
*B2: H–L–V	46000/8000/4100	8.837	10.725	857/2/105	2/0/16	811
*C3: L_CO2_	138000/24000/0	22.802	×	2748/46/2352	0/0/0	×
C4: L_CO2_	1000000/1000/0	70.490	234.196	0/0/0	0/0/0	0
C5: L_CO2_	1104000/192000/0	179.777	×	19698/303/16623	0/0/0	×
E1:2 × 2 × 2 sH	544/96/0	0.092	0.589	96/0/0	0/0/0	96
E2:4 × 4 × 4 sI	2944/512/0	0.415	1.637	512/0/0	0/0/0	512
E3:4 × 4 × 4 sII	8704/1536/0	1.160	3.082	1536/0/0	0/0/0	1536
E4:6 × 6 × 6 sII	29376/5184/0	4.152	6.072	5184/0/0	0/0/0	5184
E5:10 × 10 × 10 sH	68000/12000/0	13.178	×	12000/0/0	0/0/0	×
E6:14 × 14 × 14 sI	126224/21952/0	18.574	16.857	21952/0/0	0/0/0	21952
E7:13 × 13 × 13 sII	298792/52728/0	45.684	36.153	52728/0/0	0/0/0	52728
E8:20 × 20 × 20 sII	1088000/192000/0	172.472	138.507	192000/0/0	0/0/0	192000

a Systems marked with an asterisk (*) are from actual MD simulations; unmarked systems are supercells generated by replicating equilibrated smaller systems. The systems are labeled as L for the H_2_O liquid phase, L_CO2_ for the CO_2_ solution phase, H for the hydrate phase, and V for the CO_2_ vapor phase. Entries marked with a cross (×) indicate test cases where the reference implementation failed to complete the analysis due to execution errors.

For comparison, we have included the results from the HTR algorithm as reference, for which a publicly available executable testing version (β) is available.^47^ The HTR β version is capable of identifying 15 distinct types of standard edge-saturated cages (SECs). To ensure accuracy and computational efficiency, we selected the smallest HTR block size with a minimum threshold of 10 Å until cage number convergence was reached. The GRADE method was not included due to known limitations in reliably identifying structure I (sI) and structure II (sII) perfect hydrates and its restricted cage type coverage.^35^ The FSICA and ICO methods were also excluded because no open-source program is available for benchmarking.^34,36^

For a fair and consistent comparison, TRACE was configured to allow ring detection up to 12-membered cycles. To avoid missing some unconventional cages, the default hydrogen bond criteria were slightly relaxed, with a distance cutoff of 0.36 nm and an angle cutoff of 35° (a sensitivity analysis on the impact of the threshold values for defining a hydrogen bond is provided in Section 5 of Supporting Information). Dihedral angle pruning was disabled (pruning threshold set to 90°) to ensure maximal search completeness. To reduce thermal noise and enhance the clarity of cage detection, each trajectory was smoothed over three frames (with a 10 ps interval). If trajectory smoothing is not applied, a slightly more relaxed hydrogen bond criterion (*r*_cut_ = 0.37 nm, θ_cut_ = 40°) is recommended to accommodate greater thermal fluctuation. A sensitivity analysis of the dihedral angle tolerance settings, along with detailed simulation parameters, is provided in the Supporting Information.

The benchmark results are summarized in Table 1. TRACE successfully identified hydrate cages across all tested systems, ranging from fully disordered to perfectly crystalline phases, demonstrating robust and consistent performance under diverse conditions. It accurately detected both standard edge-saturated cages (SECs) and nonstandard or incomplete structures. It also identifies urea-coordinated SECs, non-SEC, and ICs. On the other hand, the HTR algorithm performed well in large, ordered crystalline systems. However, in certain more disordered configurations, it did not complete the analysis—possibly due to execution limitations in those settings (marked with × in Table 1). While HTR is well-suited for crystalline systems, its performance in structurally heterogeneous environments showed greater variability.

Figure 15 shows a representative frame from the simulation (model C1), illustrating the results of cage identification by both TRACE and HTR. As can be seen, TRACE not only finds more types of standard edge-saturated cages (SECs) than HTR (20 vs 12) but also more cages for each type of SEC in most cases. It is noteworthy that the public HTR version (β) used here may not be as accurate as the full HTR version, which is unfortunately not available to us.

**Figure 15:**
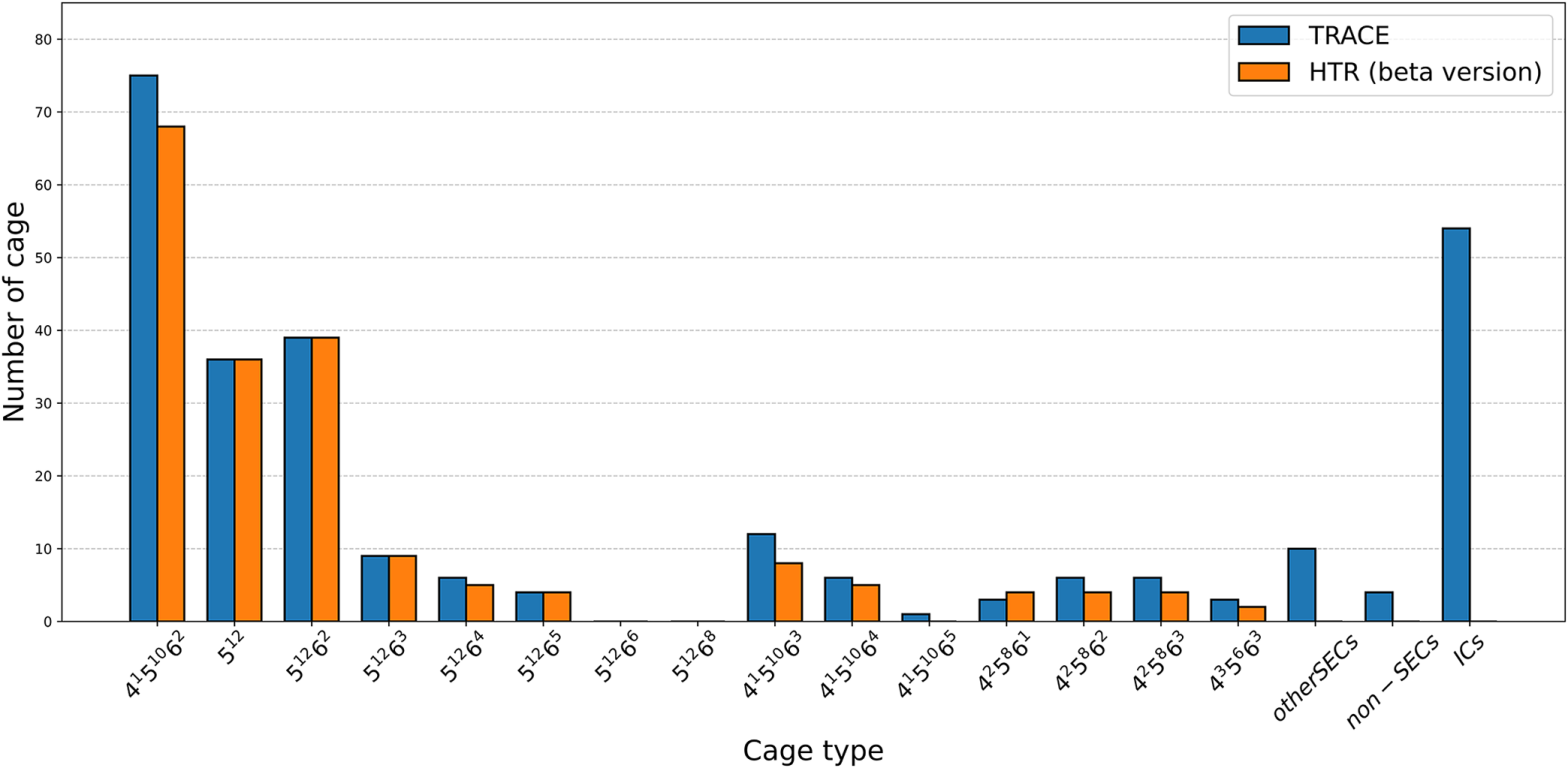
Comparison of standard edge-saturated cages (SECs), nonstandard edge-saturated cages (NSECs), and incomplete cages (IC) identified by TRACE (blue bars) and HTR (orange bars), respectively, for a representative frame from a CO_2_ hydrate nucleation simulation (model C1; 270 K, 2500 bar).

We also compared the computational performance of TRACE and HTR across perfect hydrate systems with varying numbers of water molecules (model E1 to E8) in Figure 16. In addition to detection efficiency, both TRACE and HTR demonstrated excellent computational scalability, with runtimes generally within the same order of magnitude. For small systems (<30,000 molecules), TRACE was 1.5–5 times faster, while for medium systems (60,000–300,000 molecules), HTR was slightly faster by 1.1–1.5 times. For large systems (>1,000,000 molecules), HTR maintained a modest advantage of approximately 1.24 times. Notably, although HTR is slightly more efficient for large-scale systems, TRACE—with all its features fully enabled, including comprehensive recognition of amorphous systems, tracking of all cage members, identification of incomplete cages (ICs), and detection of larger-membered rings—still maintains competitive performance, as discussed previously.

**Figure 16:**
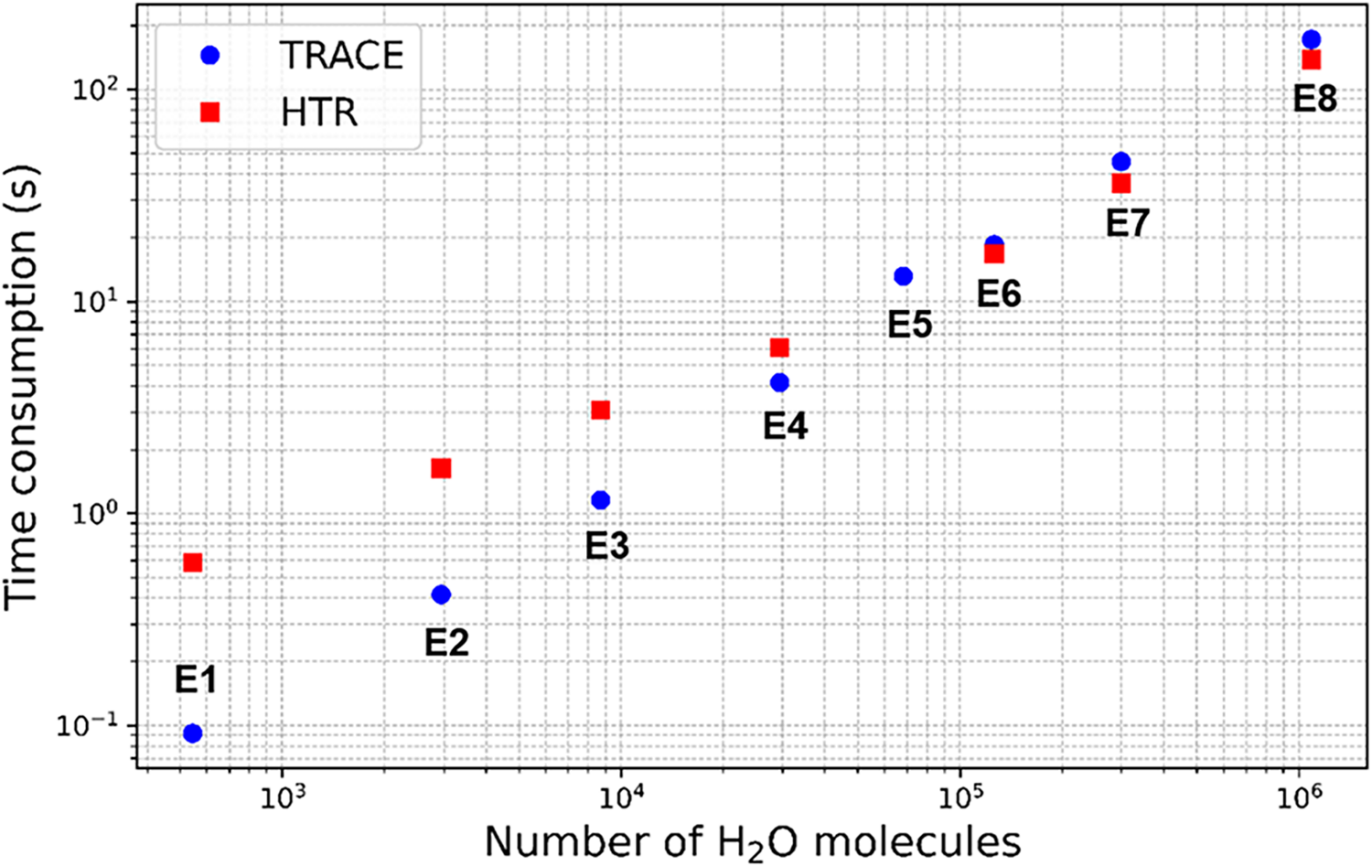
Scaling of computation time with system size for TRACE and HTR algorithms (models E1 to E8). Both axes are shown on a logarithmic scale.

To evaluate the accuracy and efficiency of the cage occupancy determination, we used the ideal sI, sII, and SH hydrate structures (model E) and achieved 100% accuracy. Using a 14 × 14 × 14 sI supercell (126,224 H_2_O and 21,952 CO_2_), guest detection increased computation time from 18.42 to 19.47 s—an overhead of ∼5.7%. These results confirm that the centroid-based guest identification method is both accurate and computationally efficient.

TRACE allows for tracking of structural evolution during a nucleation simulation. Figure 17 illustrates the time evolution of cage, cup, and ring structures during a 50 ns simulation of a supersaturated system containing 138,000 H_2_O and 24,000 CO_2_ molecules (model C3) at 2500 bar and 260 K. The results indicate that during the early stage of crystallization, the number of primary nucleation ICs significantly exceeds that of SECs and is therefore NOT negligible. Regarding the occupancy of cages, it quickly approaches a stable value close to 1 (i.e., all cages are occupied by CO_2_). As crystallization progresses, the number of 5-membered rings surpasses that of 6-membered rings, reflecting the predominance of pentagonal faces in the most stable sI, sII and SH hydrate structures. The presence of a substantial number of 7- and 8-membered rings further highlights the importance of identifying larger ring structures. In contrast, 11- and 12-membered rings are rare in systems with small guest molecules like CO_2_.

**Figure 17:**
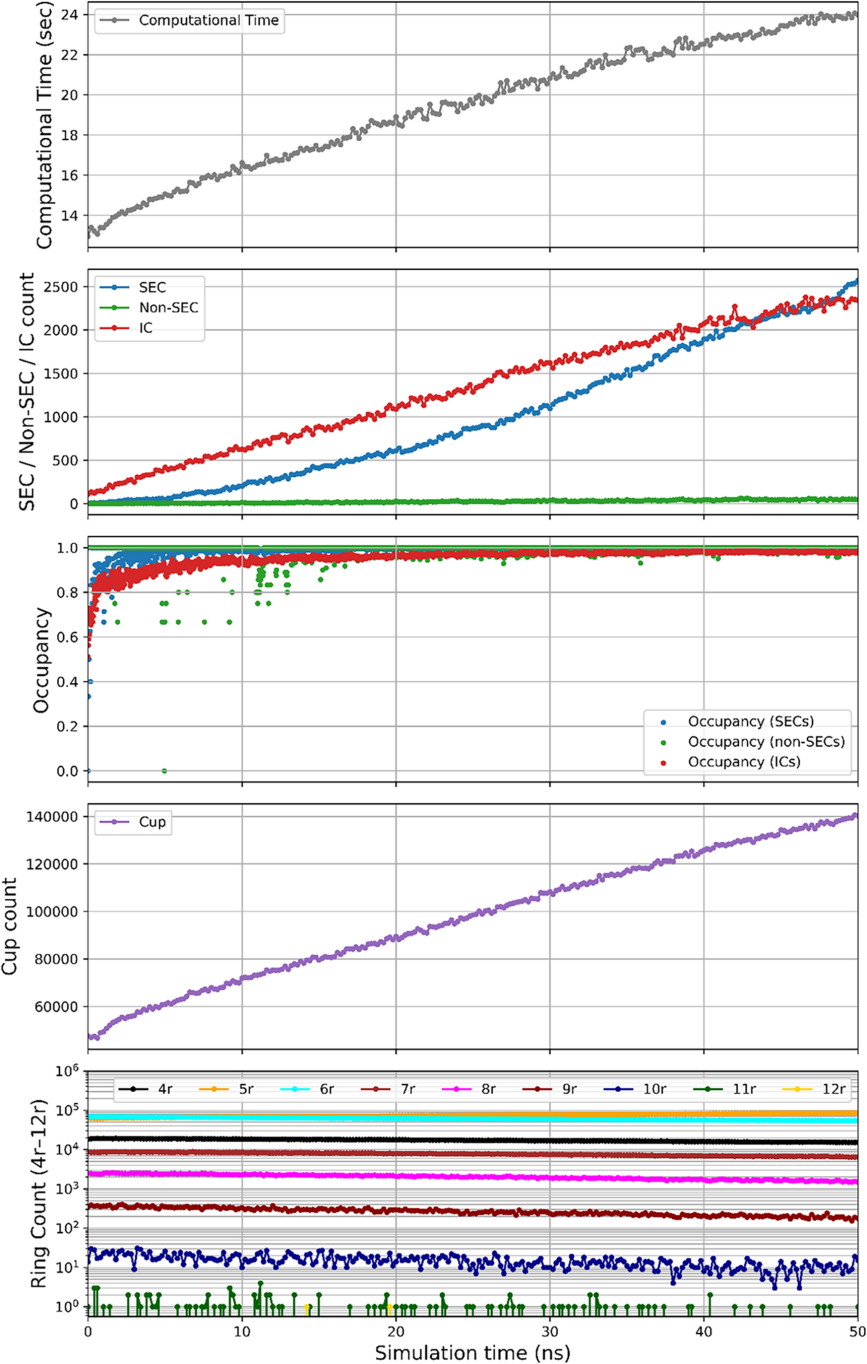
Time evolution of hydrate cage structures, cup counts, and ring distributions during a 50 ns simulation for model C3 (Table 1) at 2500 bar and 260 K.

During the induction period, a variety of intermediate motifs emerge, increasing the complexity of structural identification and resulting in a roughly linear growth in computational cost. Theoretically, once crystallization is complete, the diversity of motifs stabilizes, leading to a reduction in computational demands. For small systems (fewer than 10,000 H_2_O molecules), however, this computational overhead is minimal and can be practically ignored.

### 3.2 Analysis of Hydrate Cage Lifetimes and Occurrence Probabilities

TRACE records the molecular composition of each cage, enabling postanalysis of cage formation history. In this section, we analyze the influence of urea on the lifetimes of hydrate cages during the induction period. A total of 62 independent MD simulations were conducted for each system (with 0.66 wt % urea and without) at 257 K and 2500 bar, which is 36.5 K below the melting point of CO_2_ hydrate. The initial structures of the two systems are provided in Figure S11 of the Supporting Information. TRACE analysis shows that the urea-free system yielded 166 SEC types, 250 non-SEC types, and 3509 IC types, while the urea-containing system identified 168 SECs, 244 non-SECs, and 3566 ICs. Separately, we identified 46 SECs, 42 non-SECs, and 563 ICs as urea–coordinated cages.

Figure 18 illustrates the size evolution of the largest cage cluster in the system. The number of cages in the largest cage cluster fluctuates between 0 and 10 before 605 ns. Then the cluster size quickly rises to nearly 60 at 710 ns, after which its size fluctuates around 60. The induction time (605 ns) is visually identified as the onset of rapid increase in the size of the largest cluster in the system. The segment of trajectory before the nucleation time (induction period) is used for the cage lifetime and occurrence probabilities analysis. Specifically, if a particular cage type *k* is detected at time *i* and disappears at frame *i* + *n*, its survival time is defined as τ *= n*Δ*t* (Δ*t* being the time increment of frames). In parallel, the total occurrence count for that cage type is incremented by one (*N*_*k*_(τ) + = 1). In this work, the total number of occurrences of cage type *k*, denoted as *N*_*k*_, is calculated by summing over all survival times:

6 $$N_{k}=\sum_{\tau^{\prime }=0}^{\tau^{\prime }=\tau_{\max}}N_{k}\left(\tau^{\prime }\right)$$

where τ_max_ is the largest lifetime possible. The survival probability *S*_*k*_(τ), defined as the fraction of cages of type *k* that survive for at least time τ, is given by

7 $$S_{k}\left(\tau \right)=\frac{\sum_{\tau^{\prime }=\tau }^{\tau^{\prime }=\tau_{\max}}N_{k}\left(\tau^{\prime }\right)}{N_{k}}$$

The occurrence probability of cage type *k* is then defined as

8 $$P_{k}=\frac{N_{k}}{\sum_{k}N_{k}}$$

where the denominator includes the total number of occurrences of all cage types over all survival times, serving as the normalization factor.

**Figure 18:**
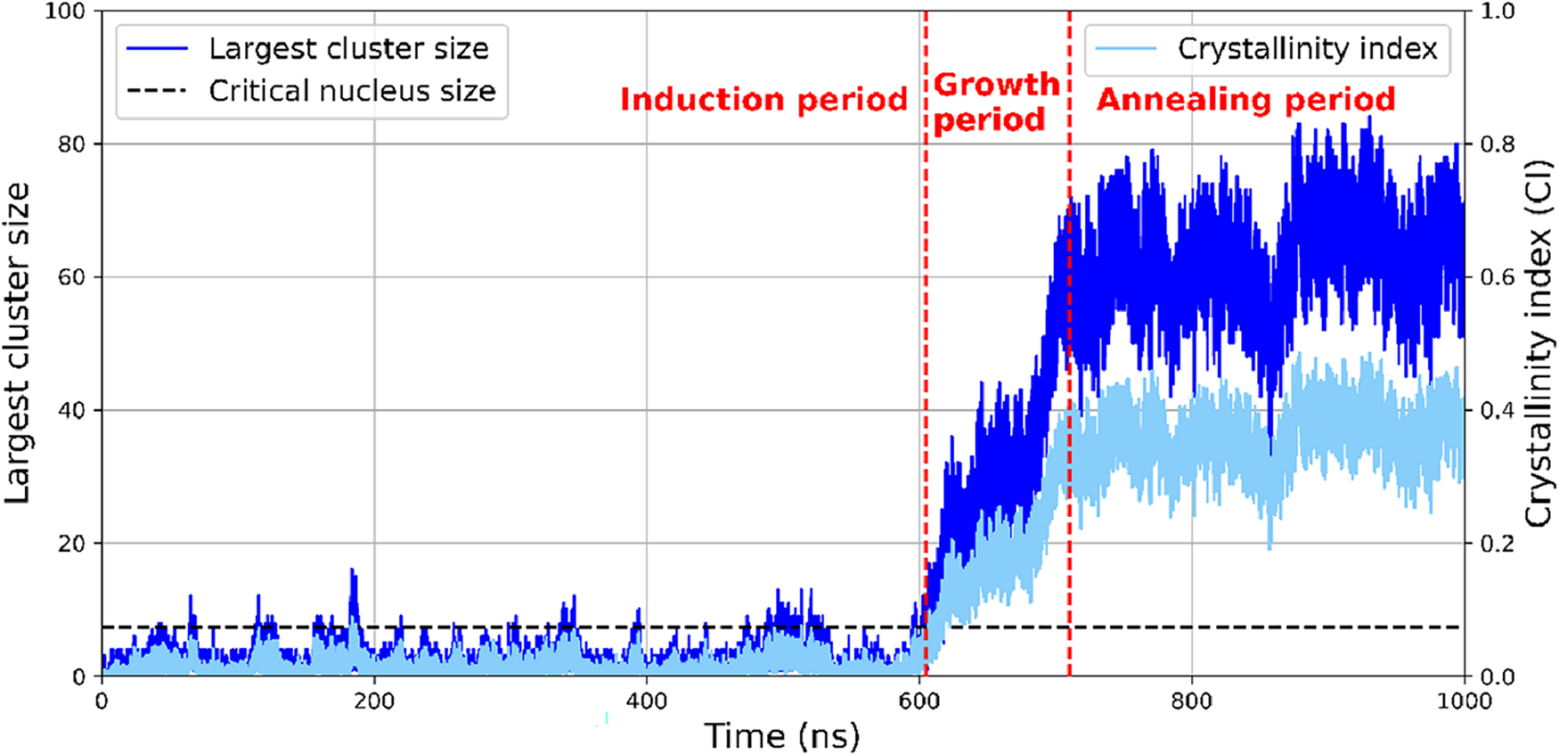
Time evolution of the crystallinity index (CI) and the number of cages in the largest cage cluster, including ICs, during CO_2_ hydrate nucleation for model A1 (257 K, 2500 bar, 0.66 wt % urea; see Table 1). The induction time, approximately 605 ns, is identified as the onset of a sustained rapid increase in the size of the largest cluster, which corresponds to the transition from the induction to the growth period. Around 710 ns, the size of the largest cluster plateaus, marking the onset of the annealing period.

The lifetime of a cage is influenced by multiple factors, such as whether it exists in isolation or within larger clusters, the presence of guest molecules at the cage center, surface adsorption of gas molecules, and its location relative to the gas–liquid interface.^48,49^ We find that the survival probability of cages formed during the induction period can be well described using a double exponential function

9 $$S_{k}\left(\tau \right)=A_{1}e^{-\tau /\tau_{1}}+A_{2}e^{-\tau /\tau_{2}}$$

where τ_1_ and τ_2_ are two characteristic cage lifetimes, and coefficients A_1_ and A_2_ are the relative populations of corresponding cage decaying dynamics. The short lifetime can be attributed to the rearrangement of molecules due to thermal fluctuations, whereas the long lifetime should correspond to the stability of cages in the induction period. Figure 19 illustrates the survival probability of 5^12^6^2^ cages from the analysis. While both the short (τ_1_) and long (τ_2_) lifetimes are reduced when urea is present (from 38.2 to 30.5 ps for τ_1_, and from 699.4 to 347.0 ps for τ_2_), the significant reduction of τ_2_ (nearly 50%) indicates the destabilization of cage structures due to urea. Similar destabilization effects can be seen for other SECs and ICs, as summarized in Supporting Tables S5–S8.

**Figure 19:**
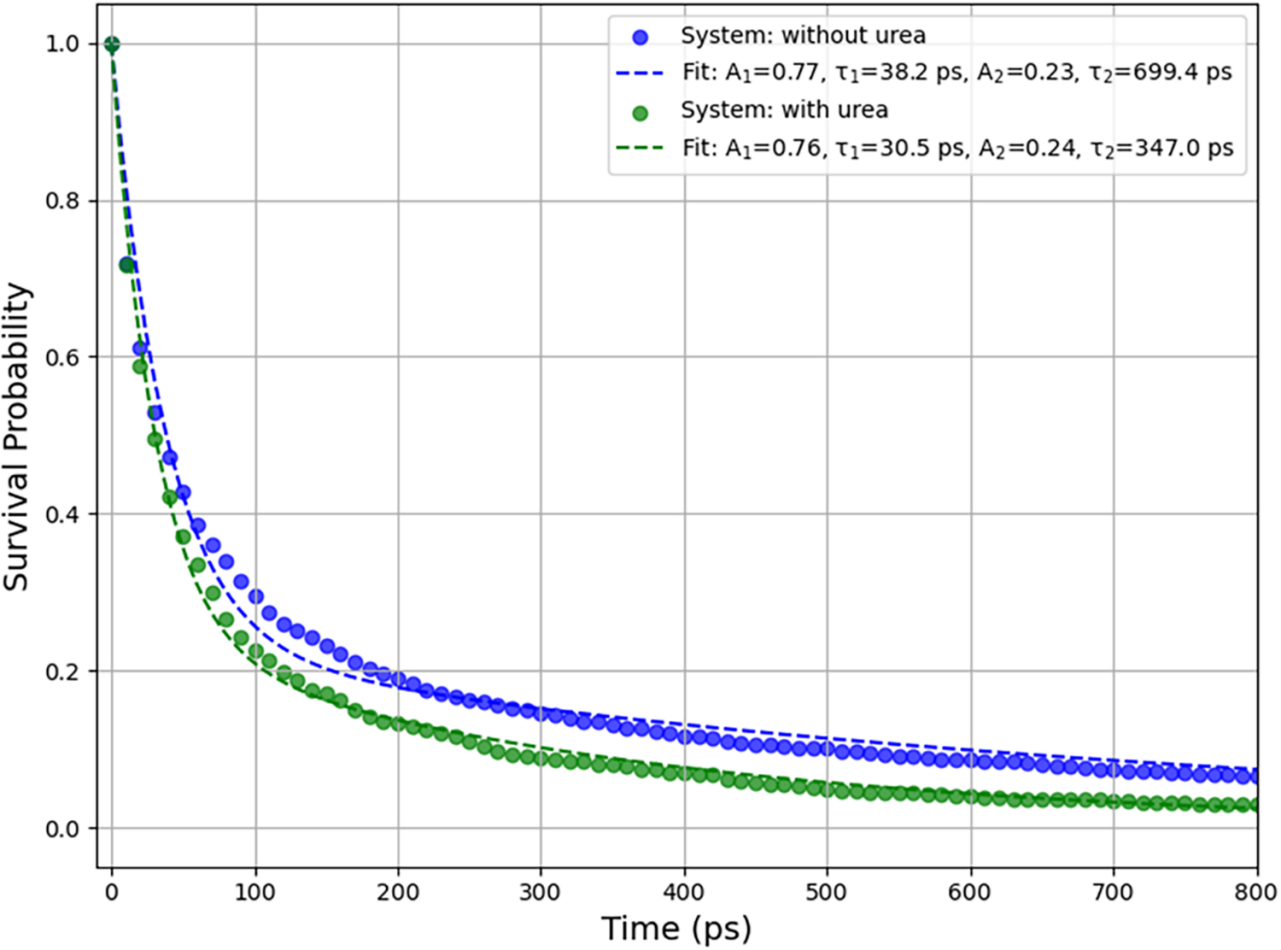
Survival probability for 5^12^6^2^ cages during the induction periods of nucleation simulations at 257 K and 2500 bar, collected from 62 independent trajectories. Systems with urea (green curve) and without urea (blue curve) are shown. The definition of the induction period is provided in Figure 18.

We further analyzed the occurrence probabilities (*P*_*k*_) of several common cage types. As shown in Figure 20, the addition of urea slightly altered the overall distribution of cage types, and a clear distinction can be observed between the distributions of water-only and urea–coordinated cages. For example, in the SECs category, urea tends to favor the formation of smaller cages such as 4^3^5^6^ and 4^2^5^8^6^1^. In the IC group, we observed several intermediate motifs resembling canonical hydrate cages like 5^12^ and 5^12^6^2^, including structures such as 5^10^6^2^ and 5^10^6^3^ (Figure 12). Notably, several of these frequently occurring cage types feature seven-membered rings. These results highlight the importance of including both ICs and seven-membered rings in the structural analysis.

**Figure 20:**
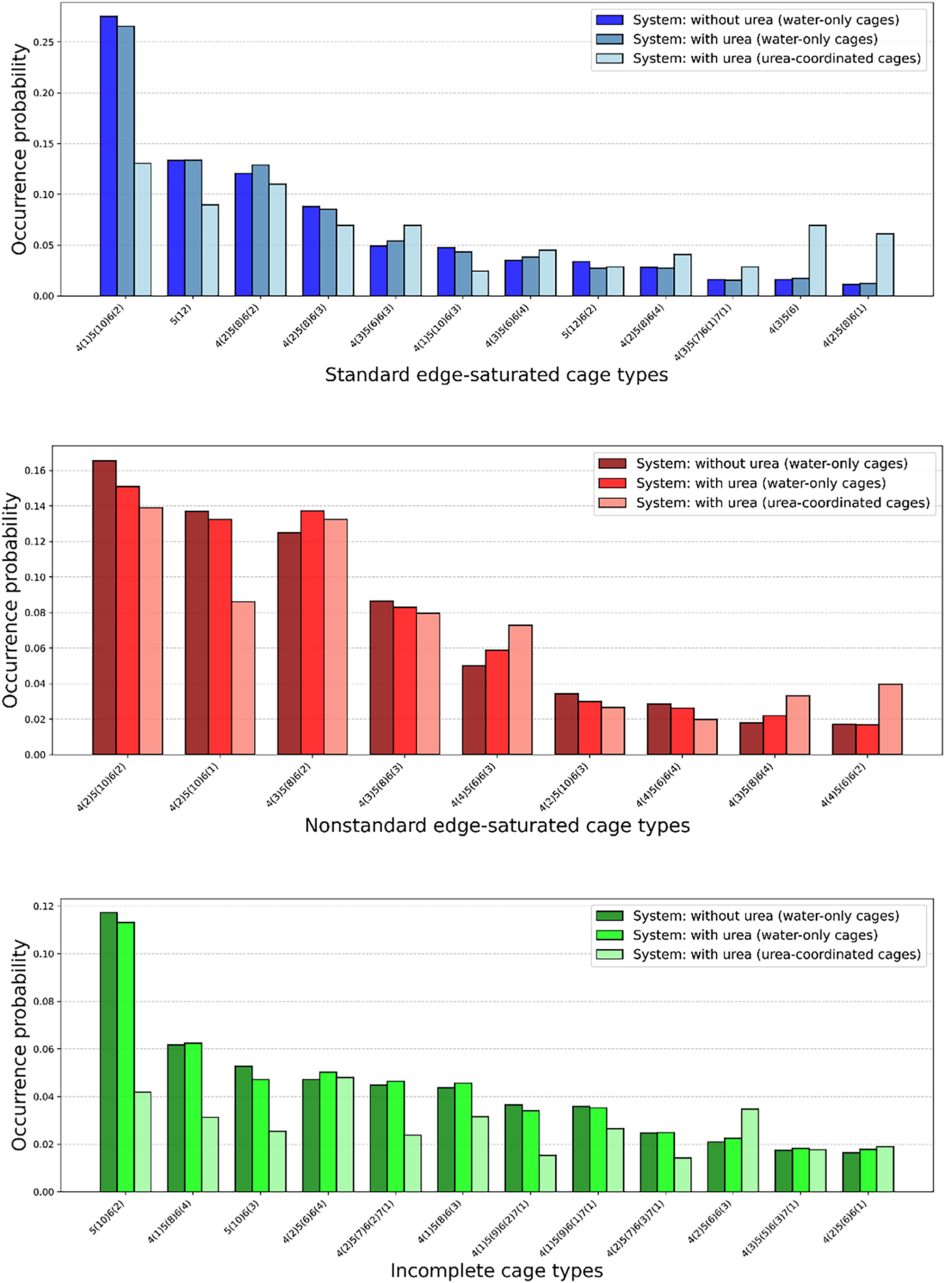
Occurrence probability of cages from 62 trajectories. Top: standard edge-saturated cages (SECs); middle: nonstandard edge-saturated cages (non-SECs); bottom: incomplete cages (ICs). Each panel shows three bins: water-only cages in pure water system, water-only cages in urea-containing systems, and urea-coordinated cages in urea-containing systems.

### 3.3 Nucleation Kinetics and Thermodynamics

The time evolution of the size of largest cage clusters from the 62 independent trajectories can be used to extract nucleation kinetic and thermodynamic parameters through the mean-first-passage-time (MFPT) analysis.^50–53^ In each trajectory, the time when the largest cluster first reaches a size *n*, τ­(*n*), is recorded, and the mean first-passage time ⟨τ­(*n*)⟩ is calculated from the average of τ­(*n*) from the 62 trajectories. Figure 21 illustrates the MFPT for the systems with urea (green circles) and without urea (blue circles). The nucleation parameters can be extracted from MFPT using the following equation^50,51^

10 $$\left\langle \tau \left(n\right)\right\rangle =\frac{\tau_{j}}{2}\left[1+\mathrm{erf}\left(\left(n-n^{*}\right)z\sqrt{\pi }\right)\right]+\frac{G^{-1}}{2}\left(n-n^{*}\right)\left[1+\mathrm{erf}\left(C\left(n-n^{*}\right)\right)\right]$$

where τ_j_ denotes the nucleation time, n* is the critical nucleus size, *z* is the Zeldovich factor, which is related to the curvature of the free energy surface. *C* is an arbitrarily large constant (set to 10,000 in this work), and *G* is a correction term accounting for the fact that nucleation and growth occur simultaneously during crystallization. The other nucleation parameters, including ΔG* (free energy barrier), γ (interfacial free energy), Δμ (chemical potential difference), and *J*_s_ (nucleation rate), can be derived from the coefficients in eq 10. Their dimensionless form is expressed as the following equation (The derivation of these parameters is discussed in Section 6 of the Supporting Information.).

11 $$\frac{{\Delta G}^{*}}{\mathrm{kT}}={3\pi n}^{*2}z^{2}$$

12 $$\frac{\gamma \rho^{-2/3}}{\mathrm{kT}}={\left(\frac{3}{32\pi }\right)}^{1/3}\Delta \mu$$

13 $$\frac{\Delta \mu }{\mathrm{kT}}=\frac{2{\Delta G}^{*}}{n^{*}}$$

14 $$J_{s}V=\frac{1}{\tau_{j}}$$

where ρ is monomer number density and *V* is system volume. The free energy Δ*G*(*n*) can be rewritten by the capillary approximation with barrier height ΔG* and critical size n*.^52,53^

15 $$\beta \Delta G\left(n\right)={\beta \Delta G}^{*}\left[3\left(\frac{n^{2/3}-1}{{n^{*}}^{2/3}}\right)-2\left(\frac{n-1}{n^{*}}\right)\right]$$

**Figure 21:**
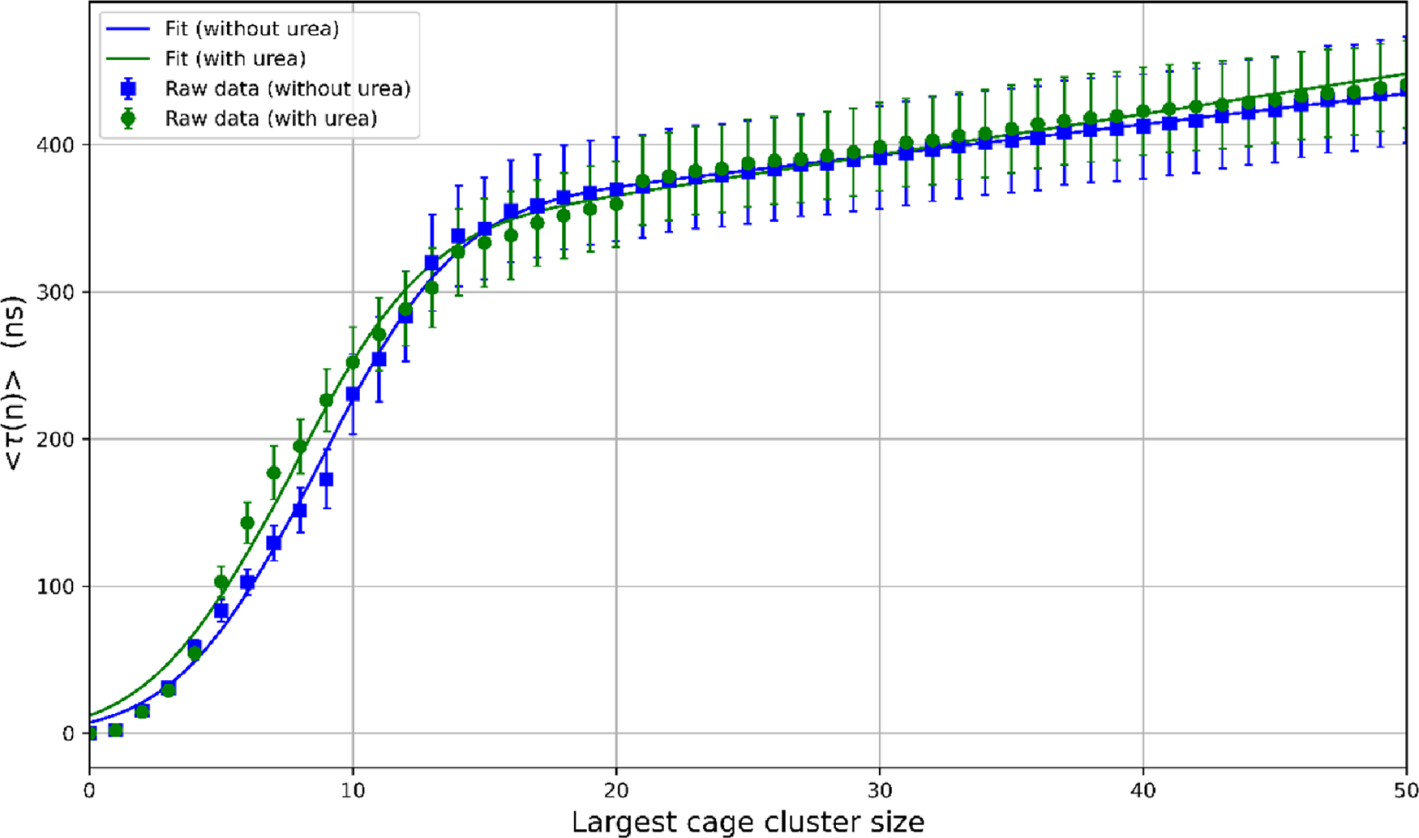
Mean first-passage time (MFPT) as a function of the largest cage cluster size at 257 K and 2500 bar, for systems with urea (green curve) and without urea (blue curve).

Table 2 summarizes and compares the nucleation parameters with and without urea present. As can be seen, the presence of urea results in a 3.5% increase in nucleation rate (*J*_s_ *V* increases from 2.9 × 10^–3^ ns^–1^ to 3.0 × 10^–3^ ns^–1^). The enhanced nucleation rate can be attributed to the reductions in the critical nucleus size n* (from 8.48 to 7.35) and barrier ΔG* (from 6.20 to 4.74 kT) and surface tension γ (from 0.925 ρ^2/3^kT to 0.778 ρ^2/3^kT). Notably, MFPT analysis revealed a decrease in chemical potential (from 1.46 to 1.29 kT), consistent with the experimental finding of urea being a thermodynamic inhibitor. In other words, the promoted nucleation by urea is a result of reduced nucleation barrier and critical nucleation size (see Figure 22).

**Figure 22:**
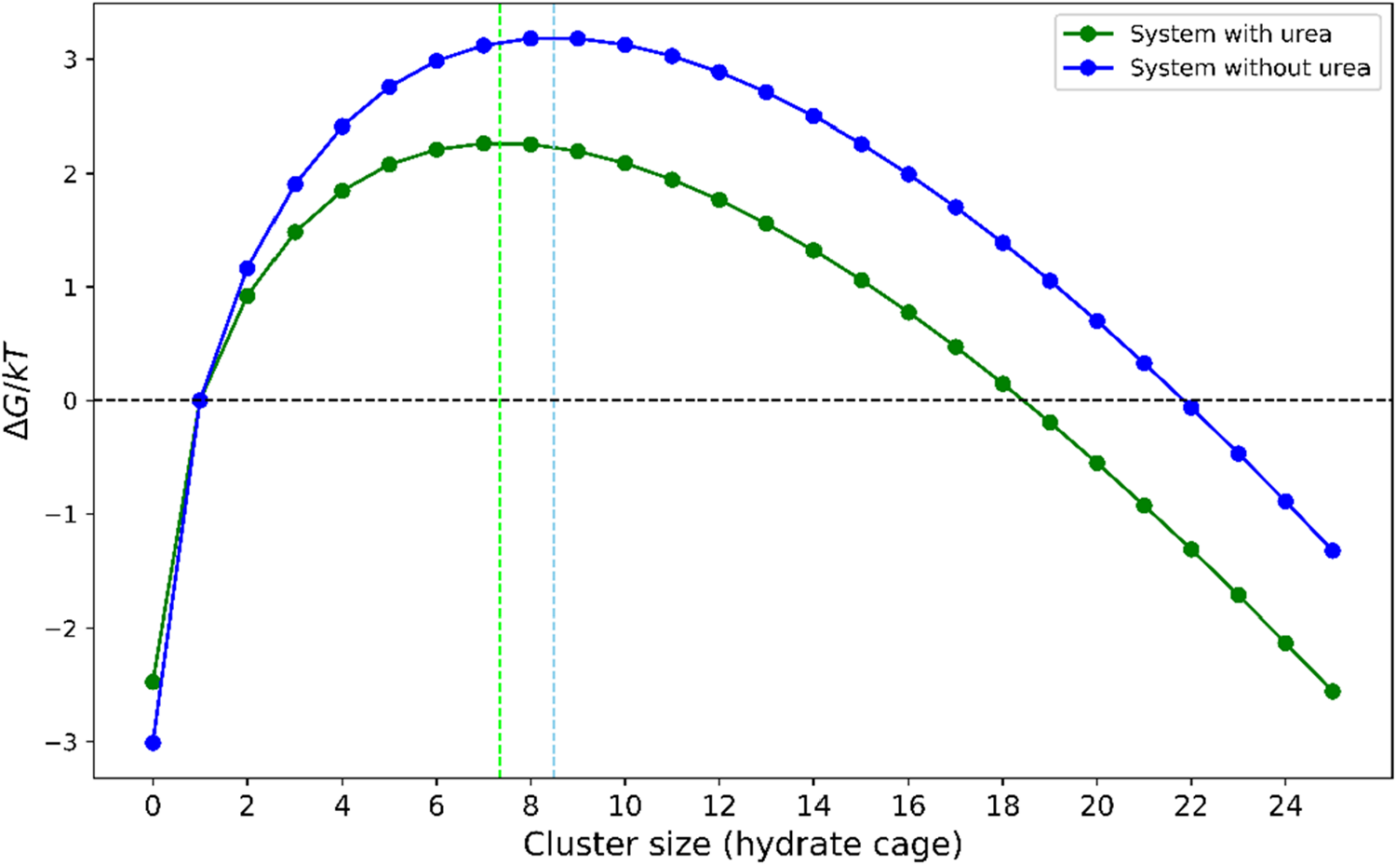
Nucleation free energy ΔG­(n) as a function of the size of cage cluster for CO_2_ hydrate nucleation at 257 K and 2500 bar with (green circles) and without (blue circles) urea.

**Table 2:** Nucleation Parameters of Systems with and without Urea (Cage Cluster)^a^

system	n*	ΔG*/kT	*J*_s_ *V* (ns^–1^)	γ ρ^–2/3^/kT	Δμ/kT
without urea	8.475	6.199	0.0029	0.925	1.462
with urea	7.350	4.742	0.0030	0.778	1.290

a n* – critical nucleus size, ΔG* – energy barrier, *J*_s_ – nucleation rate, *V* – system volume, γ – surface tension, ρ – monomer density, μ – chemical potential.

### 3.4 Dynamic Incorporation of Urea in Cages and Its Catalytic Behavior

In the previous sections, we observed that the urea molecule may participate in the cage formation and also reduces the surface tension of the hydrate nucleus. Since urea is not part of the final hydrate crystal, its presence at the nucleus-solution interface could potentially block the continuous formation of hydrate. To study why the urea-coordinated cages do not hinder the growth of hydrate, we investigate the involvement of urea at hydrate cages during the nucleation simulation.

Figure 23 shows the number of cages involving a randomly picked water molecule (molecular ID 1) and a specific urea molecule (molecular ID 1001) in the solution. The same trajectory as in Figure 18 is used here as an example. During the induction period (<605 ns), the number of cages with which water ID 1 participates fluctuates between 0 and 1, and occasionally goes up to 2, 3, and 4. Similar fluctuations in the number of cage participation can be seen for the urea molecule, except that the maximum number of cages a urea molecule participates is 2. After the induction period, the number of cage participation starts to increase for water and reaches 4 cages at the end of the simulation (1000 ns), indicating the incorporation of the water molecule into the hydrate crystal. In the case of urea, the fluctuation in cage participation increases; however, there is no clear sign of permanent incorporation of urea into the hydrate structure. The results indicate that the incorporation of urea into cage clusters is rather dynamic and does not block the growth of the cluster.

**Figure 23:**
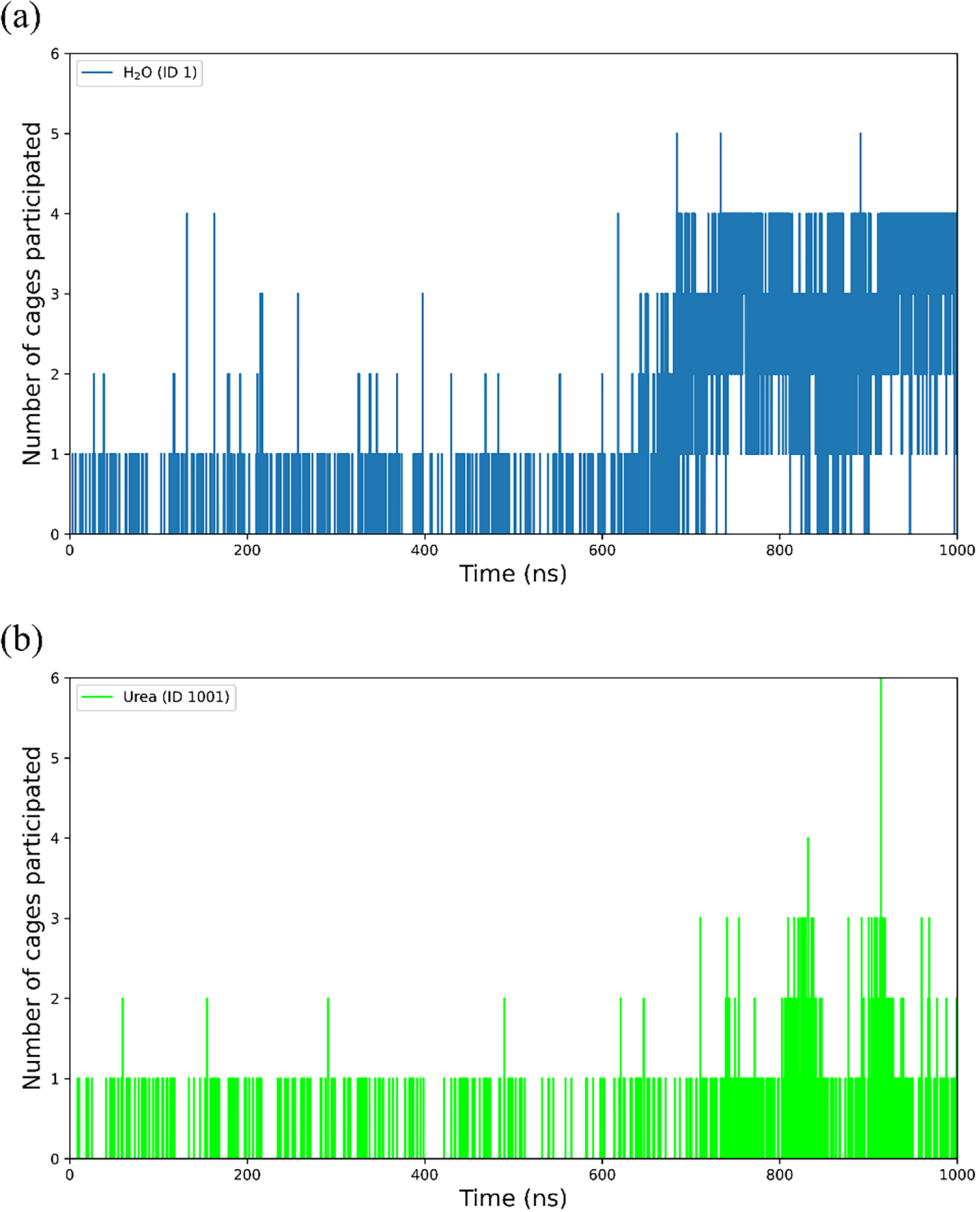
Number of cages involving a specific molecule (a) H_2_O or (b) urea during a CO_2_ hydrate nucleation simulation (model A1).

To quantify the participation of urea in the cage clusters, we analyze the retention time of molecules within hydrate cages. A retention event begins when a molecule participates in at least one cage (participation greater than zero) at frame *i*, and ends when its participation returns to zero at frame *i* plus *n*. The survival time is then defined as *n*Δ*t*, where Δ*t* is the time interval between frames. The survival probability is then calculated as *S*_*k*_ in eq 7 and modeled using a double exponential function, eq 9. Figure 24 shows the survival probability of urea incorporated in cages during different stages of nucleation: the induction period, the growth stage, and the annealing stage. As can be seen the survival probabilities are similar in the three stages. The short retention time, τ_1_ corresponds to thermal fluctuations, while τ_2_ represents longer-term retention related to cage stability. The relatively short retention time τ_2_ (20.5 ps during the induction period, and about 50 ps in the later stages) indicates dynamic and transient involvement of urea throughout the simulation. The retention time (on the picosecond scale), as discussed in this section, is considerably shorter than the time scale of cage growth, which is estimated to be about 23 ns per cage from the slope of MFPT in Figure 21. This suggests that urea does not remain trapped within cages to hinder their growth. Notably, we also observed that urea can act in a catalytic manner—facilitating the conversion of incomplete cages into complete ones, as illustrated in Figure 25.

**Figure 24:**
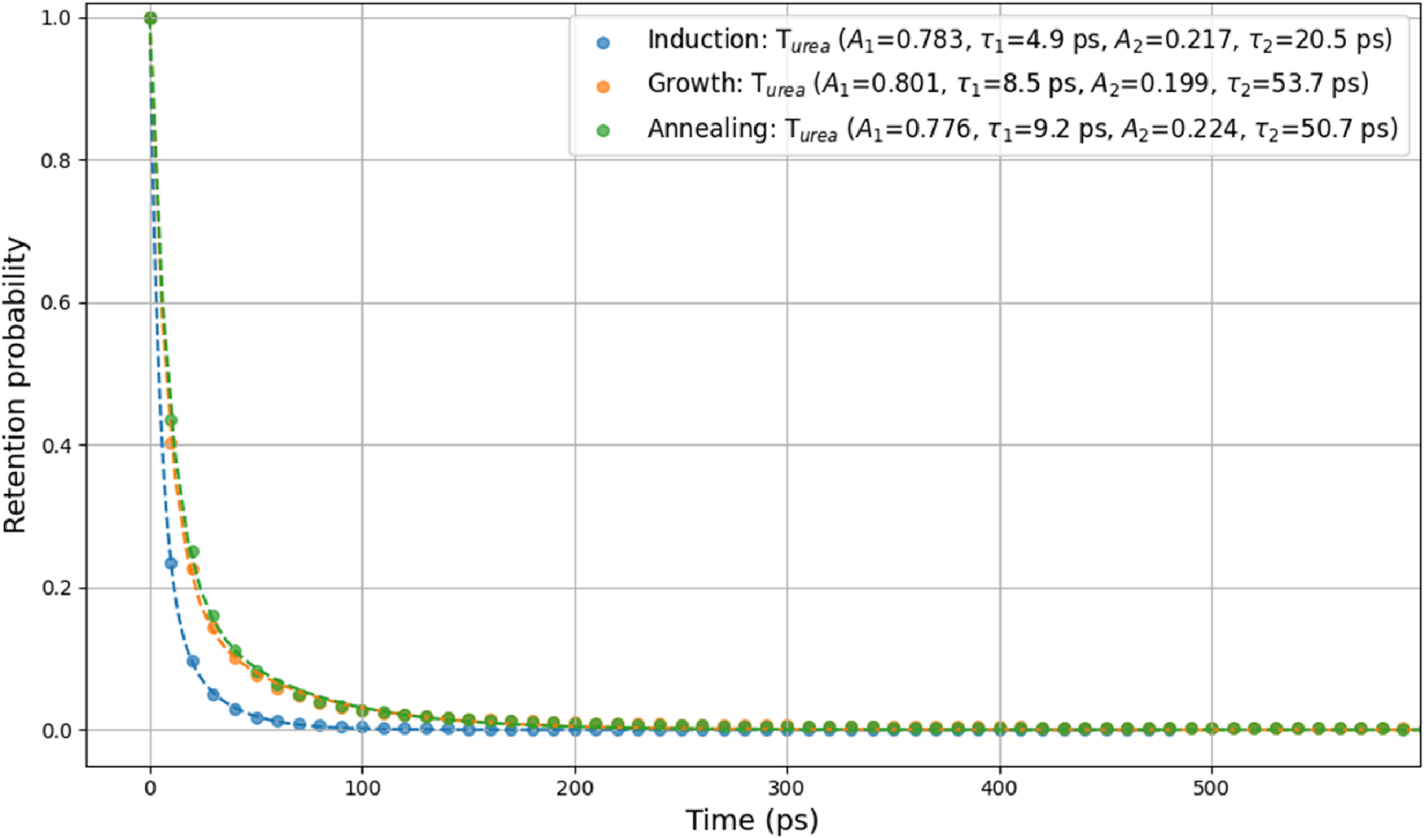
Retention time of urea molecules in hydrate cages during three periods: induction (blue circles), growth (yellow circles), and annealing (green circles) stages.

**Figure 25:**
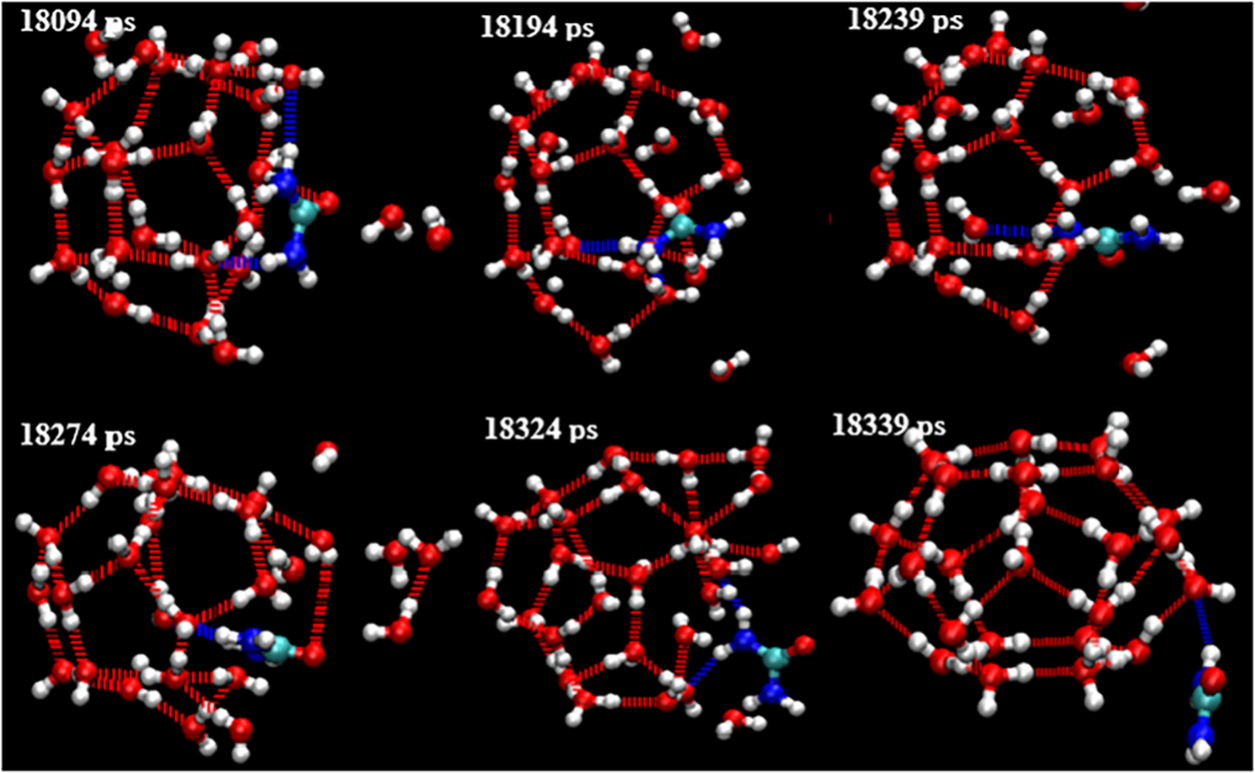
Schematic diagram illustrates how urea induces the transformation of a 4^2^5^6^6^4^ (18094 ps) incomplete cage into a 5^12^6^2^ (18339 ps) standard edge-saturated cage.

## 4 Conclusions

This work presents TRACE, a novel open-source algorithm for comprehensive topological analysis of hydrate nucleation. TRACE identifies complex ring, cup, and cage structures, extending existing approaches to improve detection accuracy and computational efficiency for both ordered and disordered systems, including those containing additives. Benchmark tests demonstrate its robustness across various hydrate phases, including large-scale simulations with over one million molecules.

While the present study focuses on urea as a representative additive, TRACE is designed to be inherently generalizable. The algorithm treats each additive as a topological vertex, with the node position for multiatom molecules defined by the geometric average of donor or acceptor atoms involved in hydrogen bonding. This design allows TRACE to accommodate diverse additives without modification (only following the user-defined hydrogen-bond specification file). More comprehensive analyses of different additives, including systems containing multiple additives simultaneously, will be addressed in future work.

By applying TRACE to molecular dynamics trajectories of CO_2_ hydrate nucleation—with and without urea additives—we gained important insights into nucleation mechanisms and the role of urea. Intermediate motifs, particularly incomplete cages (ICs) and larger-membered rings, were found to play essential roles in the early stages of nucleation. Urea alters the spatial distribution and lifetime of hydrate cages, generally reducing cage stability. However, it can also cooperate with water molecules via hydrogen bonding to promote the transformation of incomplete cages into complete ones, revealing the role of urea during hydrate nucleation. Molecular retention analysis further shows that urea interacts with cages only transiently, typically on the picosecond time scale.

Although no strong promotion effect was observed from mean first-passage time (MFPT) analysis of the largest cage cluster—likely due to the relatively low urea concentration used in simulations (0.66 wt %) compared to typical experimental conditions (∼20 wt %) and the different subcooling conditions—the simulations show that urea reduce the critical nucleus size, nucleation barrier, and surface tension, while slightly accelerating nucleation onset. These findings offer mechanistic insight into the nuanced yet potentially impactful role of additives in hydrate formation.

Overall, TRACE provides a powerful tool for detailed structural and kinetic analysis of hydrate nucleation, enabling a deeper understanding of crystallization pathways and facilitating future investigations into additive-modulated nucleation in complex molecular systems.

## Supplementary Material



## Notes

Details of model and force fields; computational methods for ring identification; parameter sensitivity analysis of TRACE; nucleation theory calculations; and data for hydrate cage lifetime analysis (PDF)

## References

[1] Sloan E. D. (2003). Fundamental principles and applications of natural
gas hydrates. Nature.

[2] Sloan, Jr, E. D. ; Koh, C. A. Clathrate Hydrates of Natural Gases; CRC press, 2007.

[3] Chong Z. R., Yang S. H. B., Babu P., Linga P., Li X.-S. (2016). Review
of natural gas hydrates as an energy resource: Prospects and challenges. Appl. Energy.

[4] Klauda J. B., Sandler S. I. (2005). Global distribution
of methane hydrate in ocean sediment. Energy
Fuels.

[5] Song Y., Yang L., Zhao J., Liu W., Yang M., Li Y., Liu Y., Li Q. (2014). The status of natural gas hydrate
research in China: A review. Renewable Sustainable
Energy Rev..

[6] Lunine J. I., Stevenson D. J. (1985). Thermodynamics of clathrate hydrate at low and high
pressures with application to the outer solar system. Astrophys. J., Suppl. Ser..

[7] Mousis O., Chassefiere E., Holm N. G., Bouquet A., Waite J. H., Geppert W. D., Picaud S., Aikawa Y., Ali-Dib M., Charlou J.-L., Rousselot P. (2015). Methane clathrates in the solar system. Astrobiology.

[8] Bashir A., Ali M., Patil S., Aljawad M. S., Mahmoud M., Al-Shehri D., Hoteit H., Kamal M. S. (2024). Comprehensive review of CO2 geological
storage: Exploring principles, mechanisms, and prospects. Earth-Sci. Rev..

[9] Nath F., Mahmood M. N., Yousuf N. (2024). Recent advances
in CCUS: A critical
review on technologies, regulatory aspects and economics. Geoenergy Sci. Eng..

[10] Park, Y. ; Cha, M. ; Cha, J.-H. ; Shin, K. ; Lee, H. ; Park, K.-P. ; Huh, D.-G. ; Lee, H.-Y. ; Kim, S.-J. ; Lee, J. Swapping carbon dioxide for complex gas hydrate structures. In ICGH 6th International Conference on Gas Hydrates; ICGH: Vancouver, Canada, 2008.

[11] Boswell R., Schoderbek D., Collett T. S., Ohtsuki S., White M., Anderson B. J. (2017). The Ignik Sikumi field experiment, Alaska North Slope:
design, operations, and implications for CO2–CH4 exchange in
gas hydrate reservoirs. Energy Fuels.

[12] Di
Giuseppe A., Gambelli A. M. (2024). CO2 Storage in Deep Oceanic Sediments
in the form of Hydrates: Energy Evaluation and Advantages Related
to the Use of N2-Containing Mixtures. Energies.

[13] Ersland G., Husebø J., Graue A., Kvamme B. (2009). Transport and storage
of CO2 in natural gas hydrate reservoirs. Energy
Procedia.

[14] Palodkar A. V., Jana A. K. (2019). Modeling recovery of natural gas
from hydrate reservoirs
with carbon dioxide sequestration: Validation with Ignik Sikumi field
data. Sci. Rep..

[15] Sharma S., Jana A. K. (2024). A Deparameterized Clathrate Phase
Description Using
Crystallography Theory: Validating Guest-Swapping Dynamics in a Gas
Hydrate. Energy Fuels.

[16] Wilberforce T., Olabi A., Sayed E. T., Elsaid K., Abdelkareem M. A. (2021). Progress
in carbon capture technologies. Sci. Total Environ..

[17] Babu P., Nambiar A., He T., Karimi I. A., Lee J. D., Englezos P., Linga P. (2018). A review of
clathrate hydrate based
desalination to strengthen energy–water nexus. ACS Sustainable Chem. Eng..

[18] Dakkumalla M. R., Babu P., Daraboina N. (2025). Clathrate
Hydrate Desalination Technology:
A Review of Recent Progress and Future Perspectives. Energy Fuels.

[19] Muromachi S., Abe T., Maekawa T., Yamamoto Y. (2015). Phase equilibrium for clathrate hydrate
formed in methane+ water+ urea system. Fluid
Phase Equilib..

[20] Elhenawy S., Khraisheh M., Almomani F., Al-Ghouti M. A., Hassan M. K., Al-Muhtaseb Aa. (2022). Towards gas hydrate-free pipelines:
a comprehensive review of gas hydrate inhibition techniques. Energies.

[21] Ho, C.-H. ; Chen, Y.-P. Measurement of Thermodynamics and Kinetics of Carbon Dioxide Hydrate in the Presence of Urea and 1,3-Cyclohexanebis­(methylamine), Master’s thesis, National Taiwan University, 2017.

[22] Kvamme B., Selvåg J., Saeidi N., Kuznetsova T. (2018). Methanol as
a hydrate inhibitor and hydrate activator. Phys.
Chem. Chem. Phys..

[23] Lin R., Huang C., Wu C., Lu C., Yu X., Li X., Li J., Wang Y. (2024). Effects of
leucine on hydrate formation:
A combined experimental and molecular dynamics study. J. Mol. Liq..

[24] Liu X., Ren J., Chen D., Yin Z. (2022). Comparison of SDS and
L-Methionine
in promoting CO2 hydrate kinetics: Implication for hydrate-based CO2
storage. Chem. Eng. J..

[25] Sinehbaghizadeh S., Saptoro A., Naeiji P., Mohammadi A. H. (2023). Molecular
dynamics simulations to investigate the effects of organic amines
on biogas clathrate hydrate formation. J. Mol.
Liq..

[26] Sinehbaghizadeh S., Saptoro A., Naeiji P., Tiong A. N. T., Mohammadi A. H. (2022). Insights
into the synergistic effects of metal particles (Ag, Cu, and Fe) and
urea on CO2 clathrate hydrate growth using molecular dynamics simulations. Chem. Eng. Sci..

[27] Sowa B., Zhang X. H., Kozielski K. A., Hartley P. G., Dunstan D. E., Maeda N. (2015). Nucleation probability
distributions of methane–propane mixed
gas hydrates in salt solutions and urea. Energy
Fuels.

[28] Sun Z., Zhou L. (2022). Clathrate hydrate formation
and crystal growth with additives. J. Mol. Liq..

[29] Wang P.-W., Wu D. T., Lin S.-T. (2021). Promotion
mechanism for the growth
of CO 2 hydrate with urea using molecular dynamics simulations. Chem. Commun..

[30] Zhang J., Lee J. W. (2009). Enhanced kinetics
of CO2 hydrate formation under static
conditions. Ind. Eng. Chem. Res..

[31] Zhang Y., Bhattacharjee G., Zheng J., Linga P. (2022). Hydrogen storage as
clathrate hydrates in the presence of 1, 3-dioxolane as a dual-function
promoter. Chem. Eng. J..

[32] Rodger P., Forester T., Smith W. (1996). Simulations
of the methane hydrate/methane
gas interface near hydrate forming conditions conditions. Fluid Phase Equilib..

[33] Barnes B. C., Beckham G. T., Wu D. T., Sum A. K. (2014). Two-component order
parameter for quantifying clathrate hydrate nucleation and growth. J. Chem. Phys..

[34] Guo G.-J., Zhang Y.-G., Liu C.-J., Li K.-H. (2011). Using the face-saturated
incomplete cage analysis to quantify the cage compositions and cage
linking structures of amorphous phase hydrates. Phys. Chem. Chem. Phys..

[35] Mahmoudinobar F., Dias C. L. (2019). GRADE: A code to
determine clathrate hydrate structures. Comput.
Phys. Commun..

[36] Hao Y., Xu Z., Du S., Yang X., Ding T., Wang B., Xu J., Zhang J., Yin H. (2021). Iterative
cup overlapping: an efficient
identification algorithm for cage structures of amorphous phase hydrates. J. Phys. Chem. B.

[37] Nguyen A. H., Molinero V. (2015). Identification of clathrate
hydrates, hexagonal ice,
cubic ice, and liquid water in simulations: The CHILL+ algorithm. J. Phys. Chem. B.

[38] Liu Y., Xu K., Xu Y., Liu J., Wu J., Zhang Z. (2022). HTR: An ultra-high
speed algorithm for cage recognition of clathrate hydrates. Nanotechnol. Rev..

[39] Shi Q., Lin Z., Qu Y., Wu J., Zhang Z. (2024). HTR+: a novel algorithm
for identifying type and polycrystal of gas hydrates. J. Phys.: Condens. Matter.

[40] Zeng J., Liu Y., Wu J., Zhang Z. (2022). Cage recognition algorithms of clathrate
hydrate and their applications. J. Cryst. Growth.

[41] Jacobson L. C., Hujo W., Molinero V. (2010). Amorphous
Precursors in the Nucleation
of Clathrate Hydrates. J. Am. Chem. Soc..

[42] Guo G.-J., Li M., Zhang Y., Wu C. (2009). Why can water cages adsorb aqueous
methane? A potential of mean force calculation on hydrate nucleation
mechanisms. Phys. Chem. Chem. Phys..

[43] Hsu, J.-W. L. ; TRACE, S.-T. URL. https://github.com/junweihsu/TRACE. 2025.

[44] Guo G.-J., Zhang Y.-G., Li M., Wu C.-H. (2008). Can the dodecahedral
water cluster naturally form in methane aqueous solutions? A molecular
dynamics study on the hydrate nucleation mechanisms. J. Chem. Phys..

[45] Luzar A., Chandler D. (1996). Hydrogen-bond kinetics
in liquid water. Nature.

[46] Laage D., Hynes J. (2006). A molecular jump mechanism
of water reorientation. Science.

[54] Takeuchi F., Hiratsuka M., Ohmura R., Alavi S., Sum A. K., Yasuoka K. (2013). Water pProton configurations in structures I, II, and
H clathrate hydrate unit cells. The Journal
of Chemical Physics.

[47] Liu, Y. , HTR, URL. https://github.com/XueerPiaoaPiao/HTR. 2022.

[48] Guo G.-J., Zhang Y.-G., Liu H. (2007). Effect of
methane adsorption on the
lifetime of a dodecahedral water cluster immersed in liquid water:
a molecular dynamics study on the hydrate nucleation mechanisms. J. Phys. Chem. C.

[49] Mastny E. A., Miller C. A., de Pablo J. J. (2008). The effect
of the water/methane interface
on methane hydrate cages: the potential of mean force and cage lifetimes. J. Chem. Phys..

[50] Wedekind J., Strey R., Reguera D. (2007). New method
to analyze simulations
of activated processes. J. Chem. Phys..

[51] Yuhara D., Barnes B. C., Suh D., Knott B. C., Beckham G. T., Yasuoka K., Wu D. T., Sum A. K. (2015). Nucleation rate
analysis of methane hydrate from molecular dynamics simulations. Faraday Discuss..

[52] Huang L.-Y., Lai P.-K., Lin S.-T. (2025). Kinetic analysis of low-barrier nucleation
via first-passage time distributions: A CO2 hydrate case study. J. Mol. Liq..

[53] Nicholson D. A., Rutledge G. C. (2016). Analysis of nucleation using mean
first-passage time
data from molecular dynamics simulation. J.
Chem. Phys..

