# Supplementary material for: TRACE: A Topological Algorithm for Detecting Additive-Coordinated Hydrate Cages
Source: J Chem Theory Comput. 2025 Nov 14;21(22):11436–54. doi: 10.1021/acs.jctc.5c01459 (PMC12659021; doi:10.1021/acs.jctc.5c01459)
Supplement: Supplementary file 1 [file ct5c01459_si_001.pdf]

# Supporting Information for

# TRACE: A Topological Algorithm for Detecting Additive-Coordinated Hydrate Cages

Jun-Wei Hsu and Shiang-Tai Lin*
Department of Chemical Engineering, National Taiwan University, Taipei 106319, Taiwan

## 1. Simulation Details

In this work, the TIP4P/Ice potential[1] was used for water, the EPM2 potential [2] for $CO_2$, and the OPLS-AA potential [3, 4] for urea. This choice of force field parameters has been shown to provide many hydrate properties that in good agreement with experiment, [4, 5].

Nonbonded interactions include both van der Waals and Coulomb interactions. The van der Waals interaction describes the exchange-repulsive and dispersive interactions. We used the Lennard-Jones 12-6 function to describe the van der Waals interaction in our simulation. Normally, the geometric combination rules for the Lennard-Jones parameters between different atoms are shown as Eq 1. However,the interaction between water and $CO_2$ is too weak compared to the real world. We introduce another parameter called scaling factor to adjust van der Waals force Eq 2. In this work we set both $X_{water\text{-}CO2}$ and $X_{water\text{-}urea}$ to 1.1. [6]

$$\epsilon_{ij} = \sqrt{\epsilon_{ii}\epsilon_{jj}} \quad (1)$$

$$\epsilon_{ij} = X\sqrt{\epsilon_{ii}\epsilon_{jj}} \quad (2)$$

The molecular dynamics simulation package GROMACS [7] is used for all MD simulations. The leap-frog algorithm [8] was used for integrating Newton's equation of motion with an integration time step of 1 fs. The Lennard-Jones potential energy and Coulomb energy were calculated with a cutoff of 9.5 Å. The Particle-Mesh Ewald (PME) [9] and dispersion corrections were used to correct the pressure [10]. The system temperature is controlled by nose-hoover algorithm [11] with $\tau_t$ = 1 ps and the pressure is control by Parrinello-Rahman algorithm [12] with $\tau_p$ = 10 ps. Before conducting the target simulation, we go through four preparatory steps.

(1) Use steepest descent algorithm for energy minimization.

(2) A short NVT simulation was then performed for 200 ps at 200 K to relax residual stresses and assign initial velocities to the molecules.

(3) Increasing the temperature at a rate of 0.5 K/ps to the desired temperature while simultaneously applying pressure coupling.

(4) Running the simulation at target condition for 1 ns or more longer for equilibrium.

(5) Target simulation

Table S1: Force field parameters

| Molecule | Atom | ε (kJ/mol) | σ (nm) | q (e) |
|---|---|---|---|---|
| **water** | O | 0.881949 | 0.316685 | 0 |
| | H | 0 | 0 | 0.5897 |
| | MW | 0 | 0 | -1.1794 |
| **$CO_2$** | C | 0.233865 | 0.2757 | 0.6512 |
| | O | 0.669335 | 0.3033 | -0.3256 |
| **urea** | C | 0.43932 | 0.375 | 0.142 |
| | O | 0.87864 | 0.296 | -0.390 |
| | N | 0.71128 | 0.325 | -0.542 |
| | H | 0 | 0 | 0.333 |

## 2. Simulation Model

Table S2 summarizes the simulation models and conditions used in this work. The systems are labeled as L for the $H_2O$ liquid phase, $L_{CO2}$ for the $CO_2$ solution phase, H for the hydrate phase, and V for the $CO_2$ vapor phase. The perfect clathrate hydrate structures were constructed based on Takeuchi et al [13]. In this work, the sH hydrate unit cell was converted from two hexagonal unit cells into an orthorhombic form to facilitate analysis and simulations.

Table S2: Simulation details for performance test

| System | # $H_2O$/$CO_2$/Urea | **Condition** |
|---|---|---|
| *A1: L-V | 1000/500/2 | 257 K and 2500 bar |
| *B1: H–L–V | 1840/320/0 | 282 K 45 bar |
| *C1: $L_{CO2}$ | 2944/512/0 | 270 K and 2500 bar |
| *A2: L-V | 3000/1500/50 | 257 K and 2500 bar |
| *C2: $L_{CO2}$ | 8832/1536/0 | 260 K and 2500 bar |
| *D1: L-V | 29440/5120/0 | 260 K and 2500 bar |
| *B2: H–L–V | 46000/8000/4100 | 275 K and 45 bar |
| *C3: $L_{CO2}$ | 138000/24000/0 | 260 K and 2500 bar |
| C4: $L_{CO2}$ | 1000000/1000/0 | × |
| C5: $L_{CO2}$ | 1104000/192000/0 | × |
| E1: 2×2×2 sH | 544/96/0 | × |
| E2: 4×4×4 sI | 2944/512/0 | × |
| E3: 4×4×4 sII | 8704/1536/0 | × |
| E4: 6×6×6 sII | 29376/5184/0 | × |
| E5: 10×10×10 sH | 68000/12000/0 | × |
| E6: 14×14×14 sI | 126224/21952/0 | × |
| E7: 13×13×13 sII | 298792/52728/0 | × |
| E8: 20×20×20 sII | 1088000/192000/0 | × |

Note: Systems marked with an asterisk (*) are from actual MD simulations; unmarked systems are supercells generated by replicating equilibrated smaller systems.

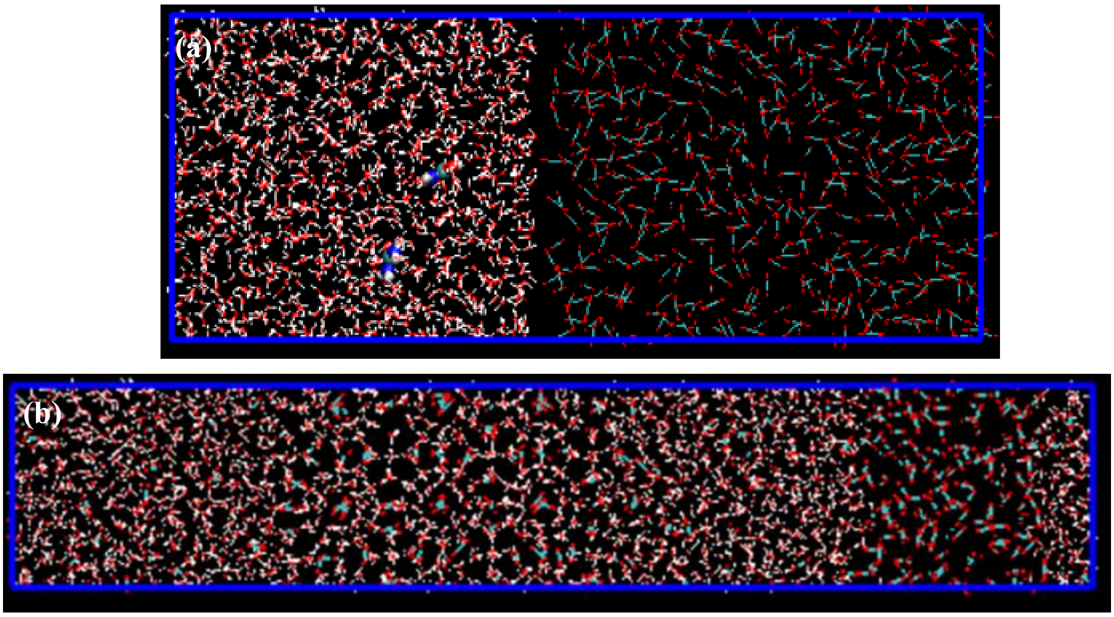


Figure S1. Representative molecular configurations of models: (a) Model A1, (b) Model B1.

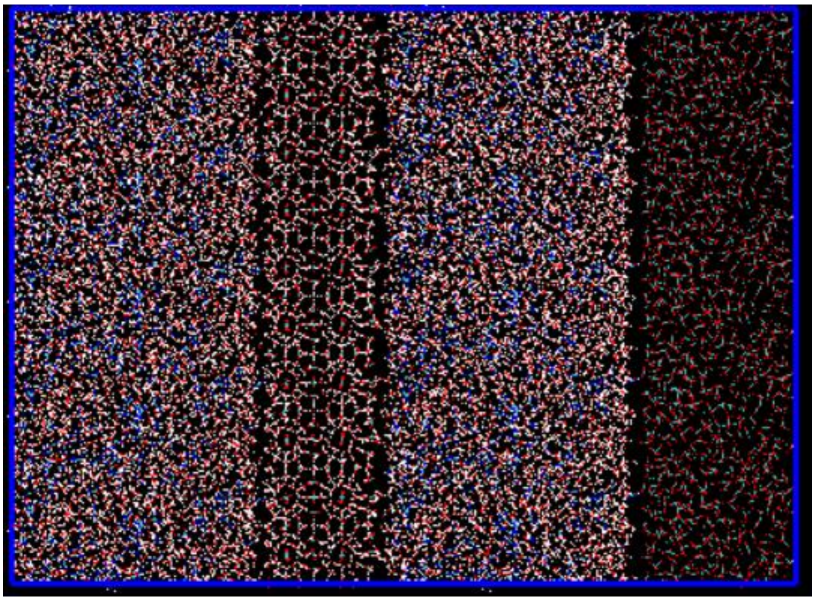

Figure S2. Representative molecular configurations of model B2

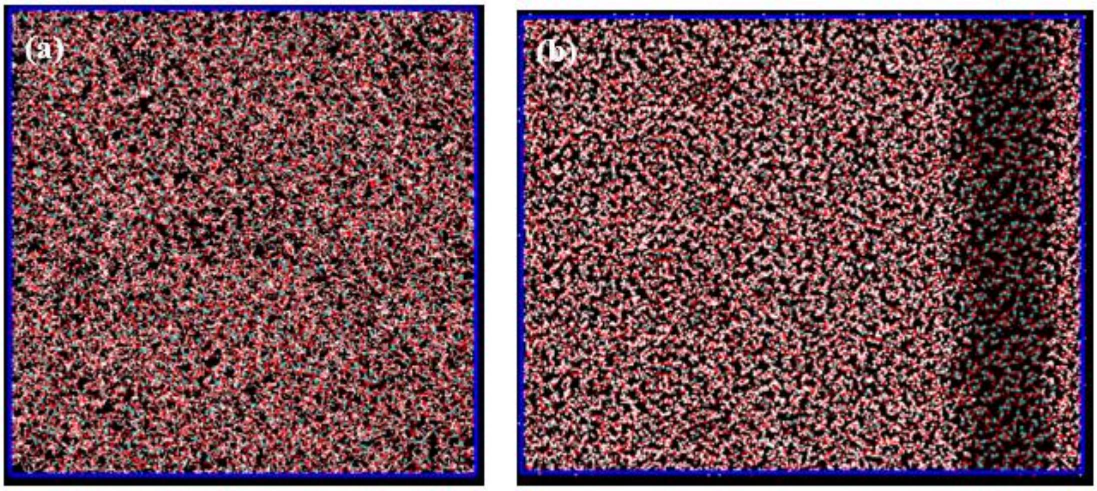


Figure S3. Representative molecular configurations of models: (a) Model C1, (b) Model D1.

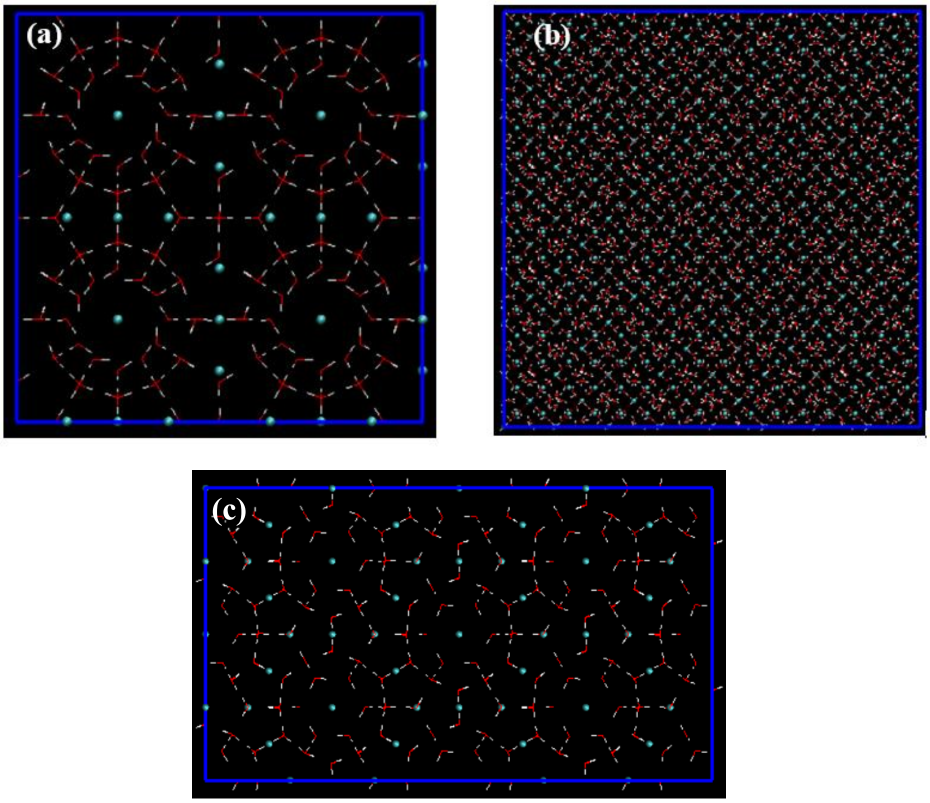


Figure S4. Representative molecular configurations of models: (a) 2×2×2 SI, (b) 4×4×4 SII (c) 2×2×2 SH (Our 1×1×1 sH model was converted from two hexagonal unit cells into an orthorhombic form. Accordingly, the figure shows a 2×2×2 system built from this converted unit).

## 3. Ring identification

One challenging aspect of ring identification is the choice of the node center when additives, such as urea, are included in the calculation of geometric properties, including interior angles, dihedral angles, or guest positions. To address this issue, TRACE records the hydrogen bond information for each hydrogen-bonding pair as a positive number corresponding to the atom indices in the .gro file (ranging from 0 to n, where 0 indicates no bond and n corresponds to the atom index). Instead of calculating the geometric center of the entire additive, TRACE computes the center based only on atoms involved in hydrogen bond formation. This approach provides a more generalizable treatment, preventing significant deviations of the node position from the actual cage or ring center that can occur with elongated additives, such as methionine.

Figure S5 shows a urea-coordinated 4-membered ring with four interior angles. During the depth-first search (DFS), each molecule is considered as a node; however, the geometric center of node 4 (the urea molecule) may vary depending on the DFS path. For $\theta_{IA,1}$ (formed by nodes 1, 2, and 4), all nodes are water molecules, so the interior angle can be directly calculated using the oxygen atoms of water molecules (atom 1, 2, and 7). For $\theta_{IA,2}$ (formed by nodes 1, 2, and 3), the water molecules are still represented by their oxygen atoms (atom 1 and 2), while node 3 (urea) uses its C=O oxygen atom (atom 6) as the center. For $\theta_{IA,3}$ (formed by nodes 2, 3, and 4), the water nodes are represented by oxygen atoms (atom 2, and 7), with the urea molecule forming hydrogen bonds with the two water molecules via its nitrogen (atom 3) and oxygen (atom 6). Thus, the geometric center of urea is taken as the average position of these two atoms. Finally, for θIA,4 (formed by nodes 1, 3, and 4), the water molecules are again represented by their oxygen atoms (atom 1 and 7), and urea is represented by its

nitrogen atom (atom 3) as the center for the angle calculation.

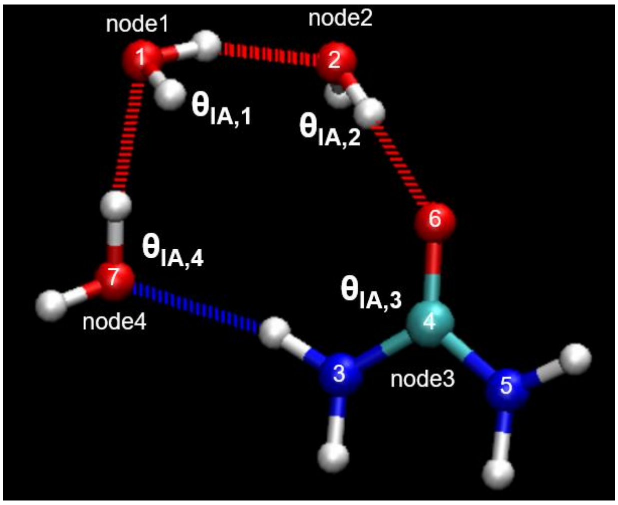


Figure S5. Urea-coordinated four-member ring.

## 4. Cage defintion in different algoritms

Table S3 summarizes the criteria used by different algorithms to identify various types of hydrate cages. Overall, the fundamental definitions of cages are consistent across methods. The primary differences lie in the number of cage types that can be detected, whether incomplete cages are recognized, and how many distinct incomplete configurations can be distinguished.

Table S3. Criteria used for identifying cages in different algorithms

| **Method** | **Complete Cage** | | **IC** |
|---|---|---|---|
| HTR | Condition 1 | | NA |
| FSICA | Condition 1 | | Condition 4 |
| GRADE | Only $5^{12}$; $5^{12}6^{2}$; and $5^{12}6^{4}$ | | NA |
| | **SEC** | **Non-SEC** | **IC** |
| ICO | Condition 2 | Condition 3 | NA |
| TRACE | Condition 2 | Condition 3 | Condition 5 |

Condition 1: meets the edge- saturation and face- saturation conditions

Condition 2: each edge is shared by exactly two faces and each vertex is shared by exactly three edges.

Condition 3: each edge is shared by exactly two faces, and each vertex is shared by at least three edges, with at least one vertex shared by more than three edges.

Condition 4: face-saturated incomplete cages (FSIC).

Condition 5: Satisfy the Euler characteristic and ensure that each edge is shared by exactly two faces (face-saturation) and each vertex is shared by at least two edges.

## 5. Parameter sensitivity analysis of TRACE

For the effects of geometric constraints on cage identification, we systematically investigated the influence of three parameters: dihedral angle tolerance, hydrogen bond angle cutoff, and hydrogen bond distance (cutoff). The tests were performed using two representative systems: an orthorhombic form of 8×8×8 sH hydrate structure containing 34,816 $H_2O$ molecules and an amorphous phase with 138,000 $H_2O$ molecules. During the analysis, only one parameter was varied at a time, while the other two were kept at their default values.

First, as shown in Figure S6, the dihedral angle tolerance significantly affects both computational cost and cage detection accuracy. For instance, in the 8×8×8 sH hydrate system, the computation time increased from 5.3 s at 30° to 6.3 s at 90°. Cage identification remains accurate in the perfect hydrate structure until the tolerance drops below 25°, beyond which the algorithm fails to detect all cages. In the amorphous phase, the number of SECs begins to converge only when the tolerance exceeds 75°, though minor fluctuations persist due to the increasing inclusion of distorted rings. Additionally, the number of ICs increases steadily with larger tolerances, indicating that relaxed

dihedral constraints allow the detection of more irregular cage-like structures.

Second, the effect of the hydrogen bond distance cutoff shows that a minimum cutoff of 0.36 nm is necessary to capture most ICs in the amorphous system (Figure S7). The number of ICs gradually converges at approximately 0.36 nm and begins to decline beyond this point. This decline is likely due to the inclusion of excessively irregular rings, which interferes with the detection of valid cages. In contrast, the perfect sH structure maintains complete cage detection even with a cutoff as low as 0.29 nm, indicating its strong structural regularity.

The influence of hydrogen bond angle constraints was evaluated using the same systems (Figure S8). In the perfect hydrate, cage detection fails below 10°, showing that overly strict thresholds exclude valid configurations. In the amorphous phase, SEC and IC counts stabilize near 45° then slightly decrease as tolerance approaches 180°, where hydrogen bond directionality is lost. This decline arises from competing effects: inclusion of nonphysical but geometrically permissible structures, and loss of valid cages due to unrealistic bonding. As shown by the $4^6$ cage example (Figure S9), removing angular constraints yields distorted yet topologically valid cages, while appropriate thresholds preserve physically realistic structures. These results highlight the importance of angular constraints to distinguish true cages from invalid ones.

Notably, when using unsmoothed trajectories, thermal fluctuations in even crystalline hydrates may require a distance cutoff of 0.37 nm and an angle threshold of 40° to ensure complete cage detection. These results highlight the need to tune constraints for different systems and trajectory processing.

Finally, we evaluated the parallel efficiency of our algorithm using a 20×20×20 sII hydrate system with 1,088,000 $H_2O$ molecules. Only parts of the algorithm, such as ring and cup detection and their sorting, are parallelizable due to the algorithm's

combinatorial and sequential nature. Our internal parallelization is applied at the level of a single frame to save memory, which inherently creates a performance bottleneck. As shown in Figure S10, speedup saturates beyond six threads, limited by contention in critical sections and the sequential steps. To better utilize CPU resources, especially if near 100% CPU utilization is desired, one can segment the trajectory into multiple frames and assign each segment to a different CPU instance, then merge the results afterward. For example, independent tasks can be run as:

$$TRACE.exe \ \ -w \ \ example.gro \ \ -b \ \ 0 \ \ -e \ \ 1000$$

$$TRACE.exe \ \ -w \ \ example.gro \ \ -b \ \ 1001 \ \ -e \ \ 2000$$

$$TRACE.exe \ \ -w \ \ example.gro \ \ -b \ \ 2001 \ \ -e \ \ 3000$$

$$\dots\dots$$

Finally, the outputs can be combined by the user, taking care to avoid overwriting since the default output file names are fixed when multiple instances are run in parallel.

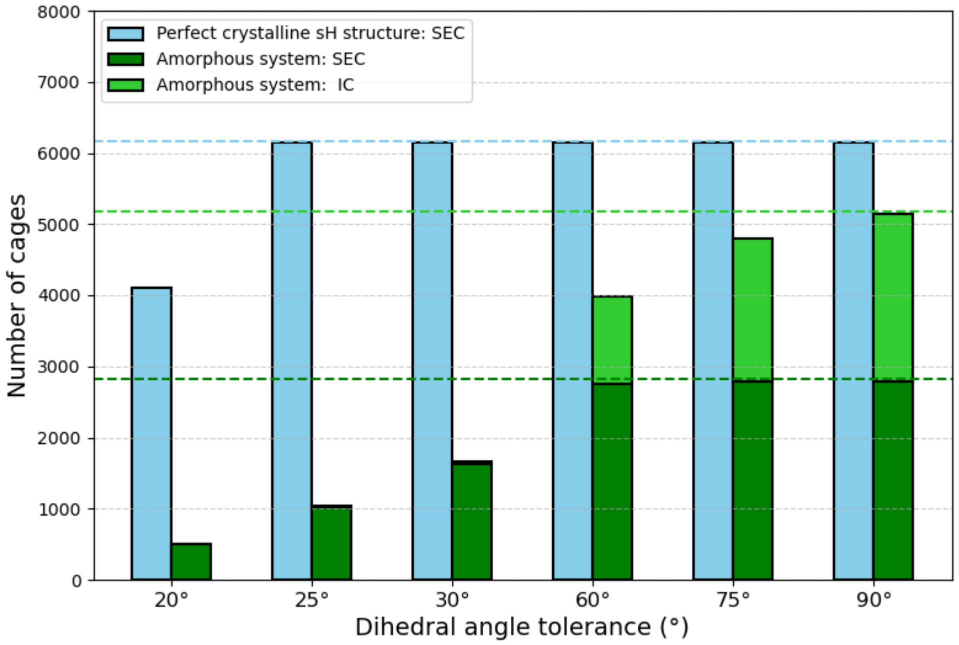


Figure S6. Variation in accuracy with dihedral angle tolerance.

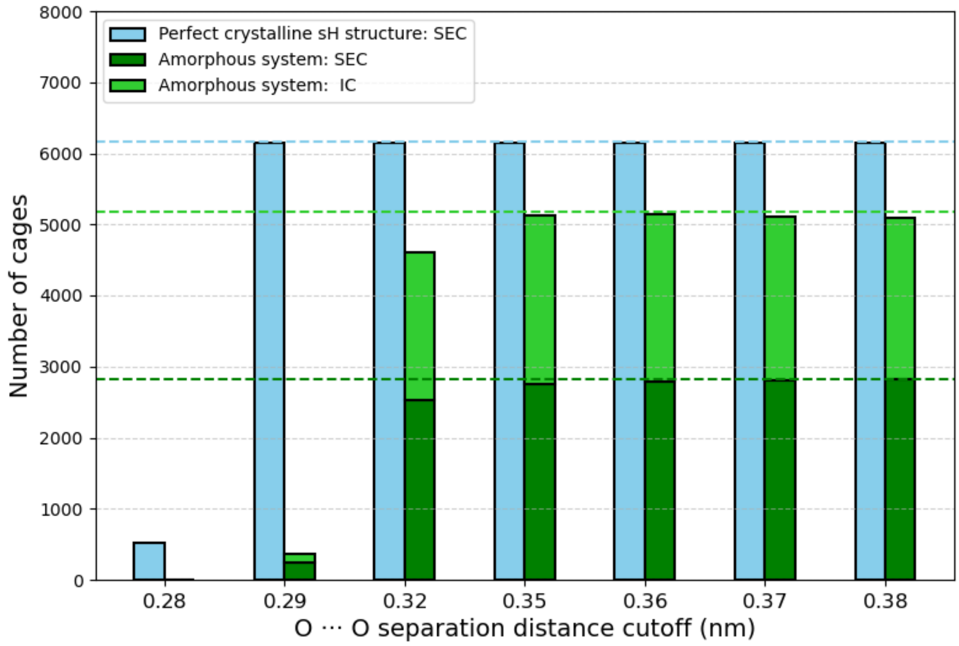


Figure S7. Variation in accuracy with h-bond distance cutoff.

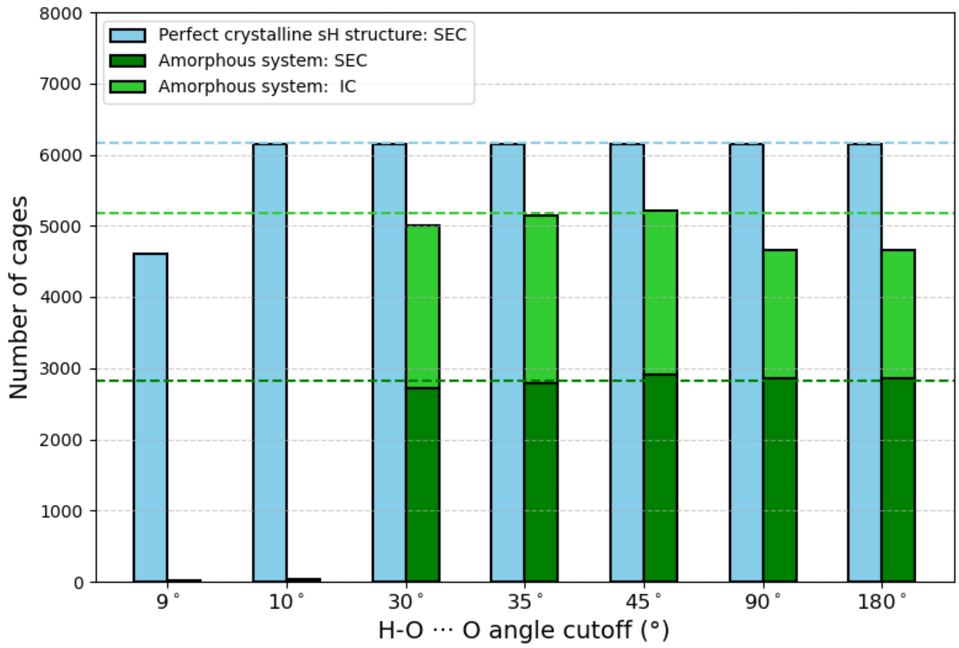


Figure S8. Variation in accuracy with h-bond angle cutoff.

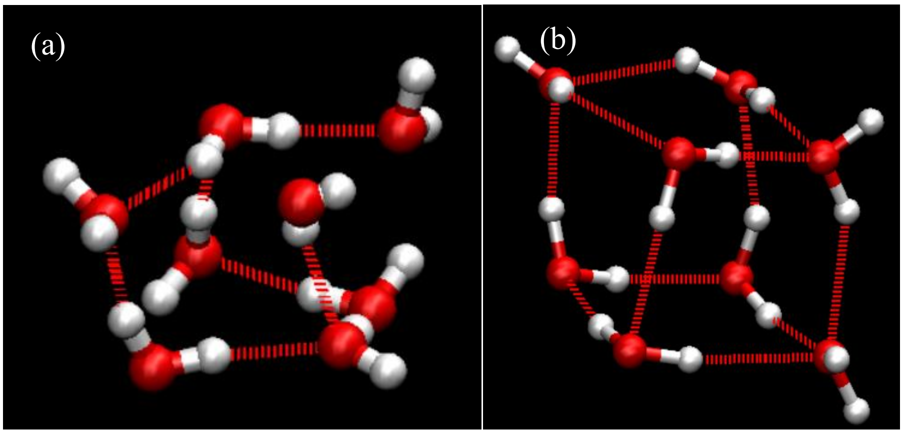


Figure S9. $4^6$ cage identification results: (a) defective structure without hydrogen bond angle filtering; (b) correct cubic structure with angle constraint applied.

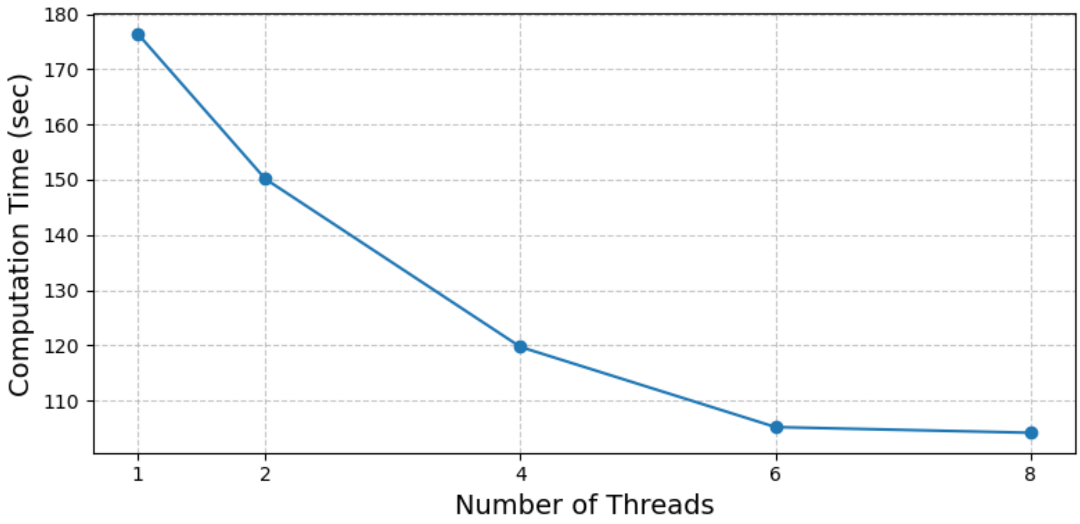


Figure S10. Parallel efficiency and scalability of cage identification algorithm on 20×20×20 S2 Structure.

## 6. Nucleation theory

For activated dynamics reaction the steady-state barrier-crossing rate can be expressed via the mean first passage time (MFPT), given by the mean first passage time (MFPT) τ [14, 15]:

$$\tau(n) = \frac{\tau_j}{2}[1 + \mathrm{erf}((n - n^*)b)] + \frac{G^{-1}}{2}(n - n^*)[1 + \mathrm{erf}(C(n - n^*))] \quad (3)$$

where G is the growth rate and C is required to be a large positive number. Then we can derive the nucleation rate $j_s$ from the $\tau_j$=1/V$j_s$. The constant b is defined as a function of the Zeldovich factor z by [14]:

$$b = \sqrt{\frac{|\Delta G''(n^*)|}{2K_BT}} = z\sqrt{\pi} \quad (4)$$

The free energy ΔG(n) can be rewritten by the capillary approximation with barrier height $\Delta G^*$ and critical size $n^*$ [16, 17].

$$\beta\Delta G(n) = \beta\Delta G^*[3\left(\frac{n^{2/3}-1}{n^{*2/3}}\right) - 2(\frac{n-1}{n^*})]$$

(5)

where β represents the inverse thermal energy, 1/$k_B$T. Note that Eq. (5) shift of the free energy reference point from n=0 (CNT convention) to n=1 (MFPT analysis) causing the nucleation barrier height $\Delta G^*$ to differ from the classical barrier $\Delta G^*$ = ΔG ($n^*$) – ΔG (0). This effect and its implications have been discussed in studies [16] . By taking the second derivative of Eq. (5) and substituting it into Eq. (4), the following expression is obtained:

$$\Delta G^* = 3 \quad \pi \quad n^{*^2} \quad z^2$$

(6)

Based on classical nucleation theory, we can derive the chemical potential difference Δμ, and the interfacial free energy γ required to create a new phase. It can be written as follows [18]:

$$\Delta G(n) = -n\Delta\mu + A(n)\gamma \ = \ -n\Delta\mu \ + \ S_m n^{2/3}\gamma$$

(7)

where *n* is the cluster size and *A(n)* is the surface area of cluster and $S_m$ is the surface area of a monomer. In this work, the monomer is defined as a single hydrate cage rather than a single molecule. Accordingly, $\rho_m$ represents the number density of cage monomers, and $S_m$ corresponds to the surface area of a single cage. We further assume that all cages are approximately spherical and have uniform density.

$$S_m = \ (36\pi)^{1/3}\rho_m{}^{-2/3} \tag{8}$$

The Gibbs free energy barrier $\Delta G^*$ occurs at the critical nucleus size $n^*$, as given by the following equation:

$$\Delta G^* = \frac{16\pi}{3}\frac{\gamma^3}{(\rho_m|\Delta\mu|)^2}$$

(9)

$$n^* = \frac{32\pi}{3}\frac{\gamma^3}{\rho_m{}^2|\Delta\mu|^3} \tag{10}$$

where $\rho_m$ is the density of monomer

By substituting Eq. (10) into Eq. (9) and rearranging, the chemical potential difference $\Delta\mu$ can be expressed as:

$$\Delta\mu = \frac{2\ \Delta G^*}{n^*}$$

(11)

By rearranging Eq. (10) yields an expression for the interfacial free energy $\gamma$:

$$\gamma = \left(\frac{3}{32\pi}\right)^{1/3} \ \Delta\mu \quad \rho_m{}^{2/3}$$

(12)

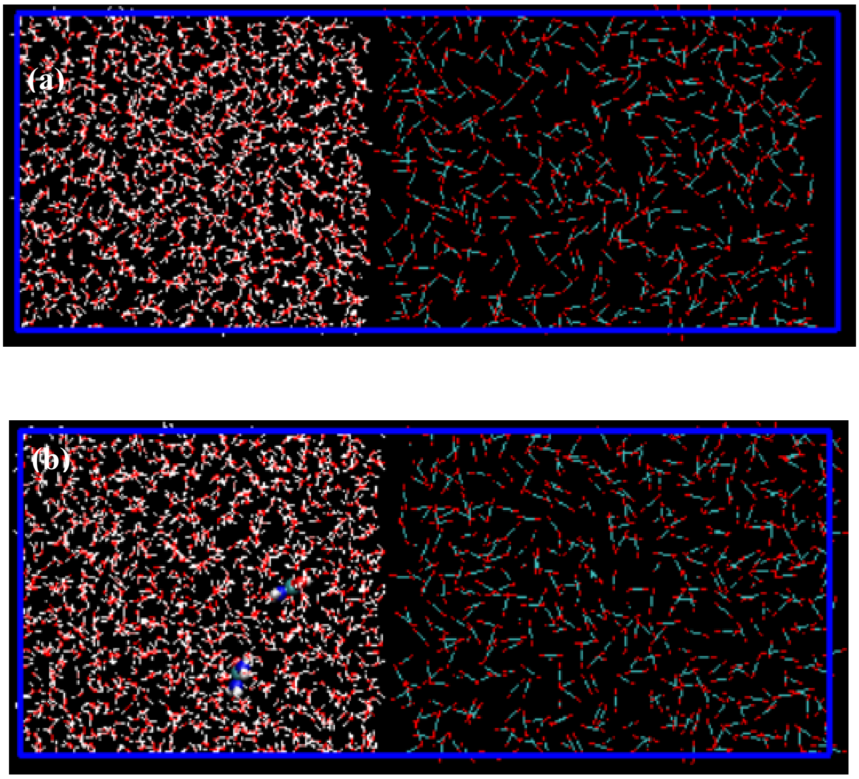


Figure S11. The initial structures used for simulation of hydrate nucleation (a) system without urea solution phase, (b) system with urea solution phase.

Table S4 Nucleation parameters of systems with and without urea (Cage cluster)

| System | GV(ns$^{-1}$) | n* | ΔG*/kT | z | $J_s$V(ns$^{-1}$) | γ ρ$^{-2/3}$/kT | μ/kT |
|---|---|---|---|---|---|---|---|
| Without urea | 0.479 | 8.475 | 6.199 | 0.096 | 0.0029 | 0.925 | 1.462 |
| with urea | 0.365 | 7.350 | 4.742 | 0.097 | 0.0030 | 0.778 | 1.290 |

Note: G-growth rate, V-system volume, $J_s$ - nucleation rate, n* - critical nucleus size, γ - surface tension, ρ - monomer density, ΔG* - energy barrier, μ - chemical potential.

## 7. Analysis of Hydrate Cage Lifetimes

This section (Tables S5–8) presents a comprehensive lifetime analysis of common

SECs and ICs, supporting the discussion in Section 3.2 of the main text. Non-SECs and urea-coordinated cages are rare events, and the 62 trajectories analyzed here are insufficient to provide statistically reliable fittings; therefore, these cases are not further discussed in this work.

Table S5: Double-exponential fit of SECs lifetimes (system without urea)

| SECs type | $A_1$ | $A_2$ | $\tau_1$ (ps) | $\tau_2$ (ps) |
|---|---|---|---|---|
| $4^1 5^{10} 6^2$ | 0.739 | 0.261 | 26.6 | 284.3 |
| $5^{12}$ | 0.723 | 0.277 | 35.2 | 379.0 |
| $4^2 5^8 6^2$ | 0.721 | 0.279 | 17.8 | 126.2 |
| $4^2 5^8 6^3$ | 0.723 | 0.277 | 17.9 | 160.3 |
| $4^3 5^6 6^3$ | 0.741 | 0.259 | 13.6 | 96.0 |
| $4^1 5^{10} 6^3$ | 0.740 | 0.260 | 23.2 | 220.5 |
| $4^3 5^6 6^4$ | 0.752 | 0.248 | 14.0 | 105.1 |
| $5^{12} 6^2$ | 0.768 | 0.232 | 38.2 | 699.4 |
| $4^2 5^8 6^4$ | 0.780 | 0.220 | 17.2 | 165.1 |
| $4^3 5^7 6^2 7^1$ | 0.664 | 0.336 | 9.3 | 57.5 |
| $4^3 5^7 6^1 7^1$ | 0.741 | 0.259 | 12.0 | 67.6 |
| $4^3 5^6$ | 0.608 | 0.392 | 5.4 | 24.9 |
| $4^2 5^8 6^1$ | 0.736 | 0.264 | 13.1 | 83.0 |
| $4^2 5^9 6^1 7^1$ | 0.449 | 0.551 | 7.3 | 51.0 |
| $4^4 5^4 6^4$ | 0.805 | 0.195 | 11.8 | 85.1 |

Table S6. Double-exponential fit of ICs lifetimes (system without urea)

| ICs type | $A_1$ | $A_2$ | $\tau_1$ (ps) | $\tau_2$ (ps) |
|---|---|---|---|---|
| $5^{10} 6^2$ | 0.653 | 0.347 | 16.7 | 91.6 |
| $4^1 5^8 6^4$ | 0.743 | 0.257 | 11.3 | 54.7 |
| $5^{10} 6^3$ | 0.677 | 0.323 | 11.6 | 56.0 |
| $4^2 5^7 6^2 7^1$ | 0.712 | 0.288 | 9.6 | 41.9 |

| | | | | |
|---|---|---|---|---|
| $4^1 6^8 6^3$ | 0.682 | 0.318 | 10.6 | 47.9 |
| $4^1 5^9 6^2 7^1$ | 0.656 | 0.344 | 8.9 | 37.8 |
| $4^1 5^9 6^1 7^1$ | 0.658 | 0.342 | 10.0 | 37.8 |
| $4^2 5^7 6^3 7^1$ | 0.683 | 0.317 | 7.9 | 31.8 |
| $4^2 5^6 6^5$ | 0.725 | 0.275 | 9.1 | 38.7 |
| $4^2 5^6 6^3$ | 0.711 | 0.289 | 8.8 | 44.5 |

Table S7. Double-exponential fit of SECs lifetimes (system with urea)

| SECs type | $A_1$ | $A_2$ | $\tau_1$ (ps) | $\tau_2$ (ps) |
|---|---|---|---|---|
| $4^1 5^{10} 6^2$ | 0.719 | 0.281 | 24.4 | 222.5 |
| $5^{12}$ | 0.680 | 0.320 | 29.5 | 293.2 |
| $4^2 5^8 6^2$ | 0.758 | 0.242 | 18.3 | 136.8 |
| $4^2 5^8 6^3$ | 0.709 | 0.291 | 15.7 | 110.5 |
| $4^3 5^6 6^3$ | 0.757 | 0.243 | 13.4 | 92.8 |
| $4^1 5^{10} 6^3$ | 0.680 | 0.320 | 18.6 | 160.5 |
| $4^3 5^6 6^4$ | 0.712 | 0.288 | 13.6 | 81.0 |
| $5^{12} 6^2$ | 0.759 | 0.241 | 30.5 | 347.0 |
| $4^2 5^8 6^4$ | 0.787 | 0.213 | 16.4 | 155.2 |
| $4^3 5^7 6^2 7^1$ | 0.637 | 0.363 | 9.9 | 46.0 |
| $4^3 5^7 6^1 7^1$ | 0.728 | 0.272 | 10.6 | 52.6 |
| $4^3 5^6$ | 0.695 | 0.305 | 5.9 | 29.4 |
| $4^2 5^8 6^1$ | 0.793 | 0.207 | 10.7 | 85.4 |
| $4^2 5^9 6^1 7^1$ | 0.615 | 0.385 | 12.8 | 64.9 |
| $4^4 5^4 6^4$ | 0.806 | 0.194 | 10.4 | 109.9 |

Table S8. Double-exponential fit of ICs lifetimes (system with urea)

| ICs type | A1 | A2 | $\tau_1$ (ps) | $\tau_2$ (ps) |
|---|---|---|---|---|
| $5^{10} 6^2$ | 0.648 | 0.352 | 15.8 | 82.6 |
| $4^1 5^8 6^4$ | 0.684 | 0.316 | 10.3 | 48.2 |
| $5^{10} 6^3$ | 0.666 | 0.334 | 11.0 | 52.4 |

| | | | | |
|---|---|---|---|---|
| $4^2 5^7 6^2 7^1$ | 0.660 | 0.340 | 9.0 | 37.0 |
| $4^1 6^8 6^3$ | 0.672 | 0.328 | 10.4 | 46.4 |
| $4^1 5^9 6^2 7^1$ | 0.692 | 0.308 | 8.9 | 38.1 |
| $4^1 5^9 6^1 7^1$ | 0.669 | 0.331 | 9.5 | 35.6 |
| $4^2 5^7 6^3 7^1$ | 0.741 | 0.259 | 8.5 | 32.1 |
| $4^2 5^6 6^5$ | 0.702 | 0.298 | 8.5 | 35.6 |
| $4^2 5^6 6^3$ | 0.711 | 0.289 | 9.4 | 47.1 |

# Reference


1. Abascal, J., et al., *A potential model for the study of ices and amorphous water: TIP4P/Ice.* The Journal of chemical physics, 2005. **122**(23).
2. Harris, J.G. and K.H. Yung, *Carbon dioxide's liquid-vapor coexistence curve and critical properties as predicted by a simple molecular model.* The Journal of Physical Chemistry, 1995. **99**(31): p. 12021-12024.
3. Duffy, E.M., D.L. Severance, and W.L. Jorgensen, *Urea: potential functions, log P, and free energy of hydration.* Israel journal of chemistry, 1993. **33**(3): p. 323-330.
4. Kokubo, H. and B.M. Pettitt, *Preferential solvation in urea solutions at different concentrations: properties from simulation studies.* The journal of physical chemistry B, 2007. **111**(19): p. 5233-5242.
5. Zhang, X., et al., *Research progress of molecular dynamics simulation on the formation-decomposition mechanism and stability of CO2 hydrate in porous media: A review.* Renewable and Sustainable Energy Reviews, 2022. **167**: p. 112820.
6. Wang, P.-W., D.T. Wu, and S.-T. Lin, *Promotion mechanism for the growth of CO 2 hydrate with urea using molecular dynamics simulations.* Chemical Communications, 2021. **57**(43): p. 5330-5333.
7. Pronk, S., et al., *GROMACS 4.5: a high-throughput and highly parallel open source molecular simulation toolkit.* Bioinformatics, 2013. **29**(7): p. 845-854.
8. Van Gunsteren, W.F. and H.J. Berendsen, *A leap-frog algorithm for stochastic dynamics.* Molecular Simulation, 1988. **1**(3): p. 173-185.

9. Toukmaji, A.Y. and J.A. Board Jr, *Ewald summation techniques in perspective: a survey.* Computer physics communications, 1996. **95**(2-3): p. 73-92.
10. Allen, M.P. and D.J. Tildesley, *Computer simulation of liquids.* Clarendon: Oxford, 1987.
11. Hoover, W.G., *Canonical dynamics: Equilibrium phase-space distributions.* Physical review A, 1985. **31**(3): p. 1695.
12. Parrinello, M. and A. Rahman, *Polymorphic transitions in single crystals: A new molecular dynamics method.* Journal of Applied physics, 1981. **52**(12): p. 7182-7190.
13. Takeuchi, F., et al., *Water proton configurations in structures I, II, and H clathrate hydrate unit cells.* The Journal of chemical physics, 2013. **138**(12).
14. Wedekind, J., R. Strey, and D. Reguera, *New method to analyze simulations of activated processes.* The Journal of chemical physics, 2007. **126**(13).
15. Yuhara, D., et al., *Nucleation rate analysis of methane hydrate from molecular dynamics simulations.* Faraday discussions, 2015. **179**: p. 463-474.
16. Huang, L.-Y., P.-K. Lai, and S.-T. Lin, *Kinetic analysis of low-barrier nucleation via first-passage time distributions: A CO2 hydrate case study.* Journal of Molecular Liquids, 2025. **418**: p. 126702.
17. Nicholson, D.A. and G.C. Rutledge, *Analysis of nucleation using mean first-passage time data from molecular dynamics simulation.* The Journal of Chemical Physics, 2016. **144**(13).
18. Kalikmanov, V.I., *Classical nucleation theory*, in *Nucleation theory*. 2012, Springer. p. 17-41.
